# Cloning of the wheat *Yr15* resistance gene sheds light on the plant tandem kinase-pseudokinase family

**DOI:** 10.1038/s41467-018-06138-9

**Published:** 2018-10-03

**Authors:** Valentina Klymiuk, Elitsur Yaniv, Lin Huang, Dina Raats, Andrii Fatiukha, Shisheng Chen, Lihua Feng, Zeev Frenkel, Tamar Krugman, Gabriel Lidzbarsky, Wei Chang, Marko J. Jääskeläinen, Christian Schudoma, Lars Paulin, Pia Laine, Harbans Bariana, Hanan Sela, Kamran Saleem, Chris Khadgi Sørensen, Mogens S. Hovmøller, Assaf Distelfeld, Boulos Chalhoub, Jorge Dubcovsky, Abraham B. Korol, Alan H. Schulman, Tzion Fahima

**Affiliations:** 1 Institute of Evolution, University of Haifa, 199 Abba-Hushi Avenue, Mt. Carmel, 3498838 Haifa, Israel; 2 Department of Evolutionary and Environmental Biology, University of Haifa, 199 Abba-Hushi Avenue, Mt. Carmel, 3498838 Haifa, Israel; 3 Institute of Biotechnology, University of Helsinki, Viikinkaari 1, P.O. Box 65, FI-00014 Helsinki, Finland; 4 Viikki Plant Science Centre, University of Helsinki, Viikinkaari 1, P.O. Box 65, FI-00014 Helsinki, Finland; 5 Triticeae Research Institute, Sichuan Agricultural University, 611130 Chengdu, China; 6 Earlham Institute, Norwich Research Park, Colney Lane, Norwich, NR4 7UZ UK; 7 Department of Plant Sciences, University of California, One Shields Avenue, Davis, CA 95616 USA; 8 The University of Sydney Plant Breeding Institute, 107 Cobbitty Road, Cobbitty, NSW 2570 Australia; 9 The Institute for Cereal Crops Improvement, Tel Aviv University, P.O. Box 39040, 6139001 Tel Aviv, Israel; 10 Department of Agroecology, Aarhus University, Forsøgsvej 1, 4200 Slagelse, Denmark; 11 School of Plant Sciences and Food Security, Tel Aviv University, P.O. Box 39040, 6139001 Tel Aviv, Israel; 12 Institute of System and Synthetic Biology—Organization and Evolution of Complex Genomes, 2 rue Gaston Crémieux CP 5708, 91057 Evry Cedex, France; 13 Howard Hughes Medical Institute, 4000 Jones Bridge Road, Chevy Chase, MD 20815 USA; 14 Natural Resources Institute Finland (Luke), Latokartanonkaari 9, FI-00790 Helsinki, Finland

## Abstract

Yellow rust, caused by *Puccinia striiformis* f. sp. *tritici* (*Pst*), is a devastating fungal disease threatening much of global wheat production. Race-specific resistance (*R*)-genes are used to control rust diseases, but the rapid emergence of virulent *Pst* races has prompted the search for a more durable resistance. Here, we report the cloning of *Yr15*, a broad-spectrum *R*-gene derived from wild emmer wheat, which encodes a putative kinase-pseudokinase protein, designated as wheat tandem kinase 1, comprising a unique *R*-gene structure in wheat. The existence of a similar gene architecture in 92 putative proteins across the plant kingdom, including the barley RPG1 and a candidate for *Ug8*, suggests that they are members of a distinct family of plant proteins, termed here tandem kinase-pseudokinases (TKPs). The presence of kinase-pseudokinase structure in both plant TKPs and the animal Janus kinases sheds light on the molecular evolution of immune responses across these two kingdoms.

## Abstract (web summary)

Yellow rust fungus severely limits global wheat production and breeding of durable resistance is challenging. Here Klymiuk et al. isolate the broad-spectrum *Yr15* resistance gene from wild emmer wheat and show that it is a member of a distinct tandem kinase-pseudokinase family of plant proteins.

## Introduction

Wheat was domesticated in the Fertile Crescent over 10,000 years ago^1^. Today, wheat is cultivated more extensively than any other food crop, covering more than 244 million ha and yielding more than 750 million tons annually (Food and Agriculture Organization Corporate Statistical Database (FAOSTAT)). It is a major component in human diet, providing starch, proteins, vitamins, dietary fiber, and phytochemicals^2^.

Wheat crops are subjected to numerous types of biotic and abiotic stresses, with fungal diseases comprising one of the most serious threats to wheat production. Yellow (stripe) rust disease of wheat is caused by the fungus *Puccinia striiformis* f. sp. *tritici* (*Pst*). Due to the rapid evolution of the pathogen and development of new virulent races, severe yield losses occurred during recent decades in Europe and worldwide^3^. Given that 88% of wheat production is susceptible to yellow rust, more than five million tons of wheat harvest, valued at around one billion USD, are estimated to be lost annually due to this pathogen^4^. These epidemics demonstrate that disease management relying on single race-specific resistance genes has become ineffective. As an alternative, broad-spectrum resistance genes are much sought after by breeders, because such genes generally can provide robust protection against diseases, in particular against yellow rust.

The plant surveillance system can detect pathogen-secreted molecules and activate powerful defense responses, which often trigger local programmed cell death (PCD) at the site of attempted colonization, known as a hypersensitive response (HR)^5^. In cereals, three different resistance mechanisms have been identified: (i) surface-localized receptors that recognize microbial-associated or pathogen-associated molecular patterns (PAMPs) and activate PAMP-triggered immunity^6^; (ii) race-specific resistance conferred by intracellular immune receptors that recognize pathogen effector proteins and activate effector-triggered immunity^6^; (iii) broad-spectrum quantitative (partial) disease resistance via various molecular pathways^7^.

Although hundreds of disease resistance (*R*)-gene loci have been genetically mapped to wheat chromosomes, only a small number of yellow rust resistance genes (*Yr*) have been isolated so far (*Yr10*, *Yr18/Lr34/Sr57*, *Yr36*, *Yr46/Lr67/Sr55*, *Yr5/YrSP*, and *Yr7*)^8,9^. Most of the cloned *R*-genes in wheat encode intracellular nucleotide-binding leucine-rich-repeat receptors (NLRs), which recognize pathogen-secreted effectors delivered into the host cytoplasm^7^. Nevertheless, among the cloned *Yr* genes only three are NLRs (*Yr10*, *Yr5*/*YrSP*, and *Yr7*), while the others represent different protein families, which act through unique mechanisms and result in broad-spectrum resistance to yellow rust (*Yr36*)^10^, and even in multi-pathogen (partial) resistance against the three wheat rusts and powdery mildew (*Yr18/Lr34/Sr57/Pm38*, *Yr46/Lr67/Sr55/Pm39*)^7^. *Yr36* encodes a wheat kinase start 1 (WKS1) protein having a non-RD kinase domain fused to a START lipid-binding domain^10^. WKS1 contributes to cell death, restricting pathogen growth and sporulation. However, this response takes several days longer than typical HR responses, thereby resulting in partial resistance^11^. *Yr46/Lr67* is a hexose transporter involved in sugar uptake^12^. Changes in two critical amino acids in the resistant allele of *Yr46/Lr67* probably alter the hexose transport in infected leaves and may explain the reduced growth of multiple biotrophic pathogen species^12^. *Yr18/Lr34* encodes an ATP-binding cassette (ABC) transporter of a yet unknown substrate^13^.

The genetic bottlenecks associated with wheat polyploidization and domestication, as well as selections in agroecosystems, led to a decrease in wheat genetic diversity and an increase in its vulnerability to biotic and abiotic stresses. One of the ways to improve wheat tolerance to these stresses is to recruit the adaptive potential of the wild wheat germplasm. Wild emmer wheat (WEW; *Triticum turgidum* ssp. *dicoccoides*), the tetraploid progenitor of hexaploid common wheat (*Triticum aestivum*), has valuable adaptive diversity to various diseases, including yellow rust^1,14^.

*Yr15* is a yellow rust resistance gene discovered in the 1980s in WEW accession G25 (G25)^15^. *Yr15* confers broad-spectrum resistance against a worldwide collection of more than 3000 genetically diverse *Pst* isolates, including modern races, such as ‘Warrior’ (race DK09/11), which is currently threatening wheat production^8,16–18^. Although some *Pst* races were reported to be virulent on *Yr15* in 2000s^19^, since 2003 no virulence on *Yr15* was reported among new incoming *Pst* isolates (Global Rust Reference Center (GRRC)).

Previously, we had mapped *Yr15* on chromosome arm 1BS distal to the nucleolar organizer locus *Nor-B1* using restriction fragment length polymorphism and simple sequence repeat markers^20,21^. The development of near isogenic lines (NILs) for *Yr15* in the common wheat Avocet background^8^ and the identification of DNA markers linked to this gene^21^ have accelerated the introgression of *Yr15* into a variety of pre-breeding and commercial materials, including cultivars, such as Clearwhite 515, Expresso, Patwin 515, and Seahawk, that are widely grown in western United States^8,21^. In light of the above, *Yr15* will find use in wheat breeding in many other wheat-growing regions^8^.

Here, we describe the identification of the *Yr15* gene sequence by map-based cloning and demonstrate its function in conferring resistance to yellow rust in durum and common wheats, by both ethyl methanesulfonate (EMS) mutagenesis of resistant *Yr15* introgression lines (ILs) and transgenic complementation of susceptible varieties. We show that *Yr15* encodes a protein composed of putative kinase and pseudokinase domains in tandem, which we name here as WTK1 (wheat tandem kinase 1). Orthologs, paralogs, and homologs of *WTK1* were found in other cereal species. Furthermore, we have discovered similar tandem kinase-pseudokinase (TKP) structures in a wide range of plant taxa and suggest that they belong to the same protein family, designated here as TKP family.

## Results

### Variation in resistance response of *Yr15* ILs

A set of *Yr15* ILs inoculated with *Pst* isolate #5006 showed a range of resistance responses, probably due to differences in their genetic backgrounds (Supplementary Fig. 1a). Infection types (ITs) for these ILs ranged from 0 (no visible response, e.g., B70 (*Yr15*)) to 2 (large chlorotic blotches, e.g., Sel46 (*Yr15*)), with clear HR visible to the naked eye in most of the *Yr15* ILs (Supplementary Fig. 1a).

Previous studies showed that although *Yr15* conferred broad-spectrum resistance against a worldwide collection of *Pst* isolates^16–18^, a few isolates that appeared in Europe more than a decade ago (e.g., isolate DK92/02) showed virulence on *Yr15* IL Avocet + *Yr15*^19^. Therefore, we have challenged a set of *Yr15*, *Yr5*, and *Yr5*/*Yr15* ILs with *Pst* races known to be virulent on *Yr15* (DK92/02)^19^ or *Yr5* (AU85569)^22^. Indeed, DK92/02 was virulent on Avocet + *Yr15* (IT = 5–7), and AU85569 was virulent on Avocet + *Yr5* (IT = 7), while both of these races were virulent (IT = 8–9) on Avocet S NIL (Supplementary Fig. 1b). However, pyramiding *Yr15* with *Yr5* in four different backgrounds (YecoraRojo*Yr5Yr15*, Patwin*Yr5Yr15*, Summit*Yr5Yr15*, and Dirkwin*Yr5Yr15*) showed full protection against both virulent isolates (Supplementary Fig. 1b).

### Mechanism of resistance associated with HR

**Fig. 1:**
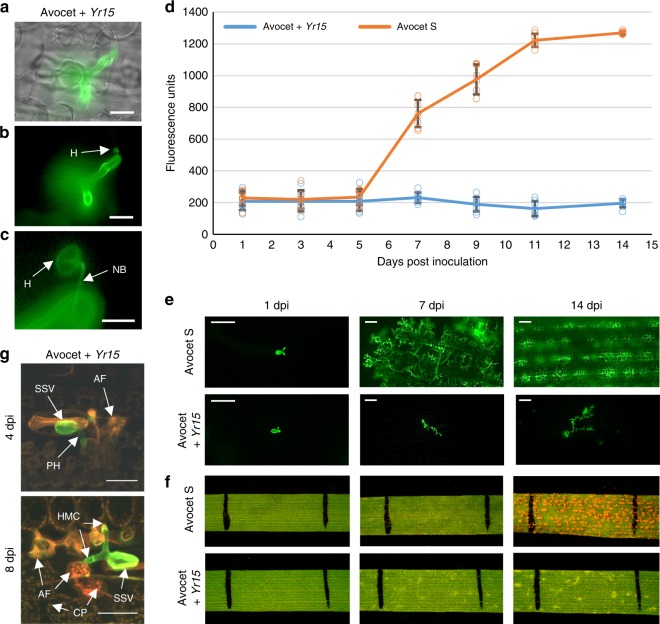
Fungal development in *Pst*-infected Avocet S and Avocet + *Yr15* NILs during 14 days post inoculation (dpi). **a**–**c** *Pst* haustorium (H) within invaded plant host cell observed in Avocet + *Yr15* at 3 dpi under bright field (**a**) and fluorescence (**b**), connected to HMC via neckband (NB; **c**). Bars = 20 µm (**a**, **b**), 5 µm (**c**). **d** Comparative amounts of fungal biomass (chitin) within leaf tissues of NILs during 1–14 dpi. Error bars denote standard deviation (s.d.) based on eight biological replicates. **e** Fluorescence micrographs of fungal colonies and feeding structures at 1, 7, and 14 dpi in susceptible and resistant NILs. Bars = 100 μm. **f** Leaf segments at 1, 7, and 14 dpi in susceptible and resistant NILs. The black lines delineate a 1 cm segment in the middle of the second leaf of the same plant. **g** Fungal structures and host responses in Avocet + *Yr15* at 4 and 8 dpi. The fungal colony (in green) consist of a substomatal vesicle (SSV) and primary infection hyphae (PH) with haustorial mother cells (HMC). Indicative signs of host cell autofluorescence (AF) are visible already at 4 dpi, while at 8 dpi the infected host cells show bright orange AF and appear to be collapsed and distorted; a couple of them contain aggregated chloroplasts (CP) with bright fluorescence as an indicator of host HR. Bars = 40 µm

Microscopic observations of the interactions between plant host cells and the invading pathogen showed the formation of fungal structures within leaf tissues, including substomatal vesicles, primary infection hyphae, haustoria mother cells (HMCs), and haustoria already at 1 day post inoculation (dpi) in both the susceptible common wheat genotype Avocet S and its resistant NIL Avocet + *Yr15* (Supplementary Fig. 2a). Moreover, high-resolution close-up images presented in Fig. 1a–c clearly show HMCs, very thin penetrating hyphae (neckband) and haustoria feeding structures inside invaded mesophyll cells of Avocet + *Yr15* at 3 dpi. Substantially larger fungal colonies and much more abundant pathogen feeding structures were observed in Avocet S compared with Avocet + *Yr15* NIL, 3–14 dpi (Fig. 1d, e and Supplementary Fig. 2a). Relative quantification of fungal development and colonization of Avocet S (Fig. 1d) fitted the standard logistic population growth model well and displayed highly significant increase in biomass (*p* < 10^–15^, likelihood ratio test), which is typical for the growth of microorganisms under favorable conditions^23^. By contrast, colonization of Avocet + *Yr15* was negligible and did not change significantly 1 to 14 dpi (*p* = 0.21, likelihood ratio test) (Fig. 1d). At 7 dpi fungal biomass residing within infected leaf tissues began to show significant differences between NILs (*p* < 0.001, *t* test, *n* = 16) (Fig. 1d), when macroscopic evidence of HR became to be visible on Avocet + *Yr15* leaves, but not on Avocet S (Fig. 1f and Supplementary Fig. 2b). The differences in fungal growth between the NILs can be attributed to the strong HR response in Avocet + *Yr15*, which restricts the fungal growth at very early stages of infection. The host cells that are interacting with fungal feeding structures show clear indications of HR, as evident from the collapse and distortion of these cells, strong autofluorescence as well as the aggregation of the chloroplasts within the infected cells (Fig. 1g and Supplementary Fig. 3a–d). These results suggest that HR plays a central role in restricting the development of fungal feeding structures.

### Map-based cloning of *Yr15*

**Fig. 2:**
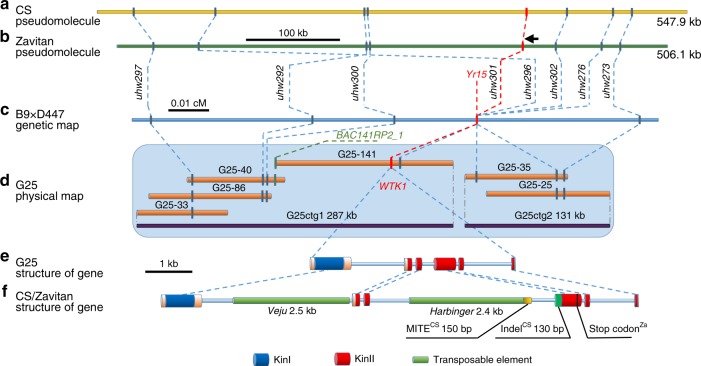
Map-based cloning of *Yr15*. **a**, **b** Genomic sequence of CS (**a**) and Zavitan (**b**) 1BS pseudomolecules harboring the *WTK1* region. **c** Genetic map of the 1BS region carrying *Yr15*. Marker *uhw301* was developed from the *WTK1* sequence. **d** G25 BAC-based physical map covering the *Yr15* region. Assembly of BAC sequences yielded contigs G25ctg1 (287 kb) and G25ctg2 (131 kb). Marker *BAC141RP2_1* was used to connect G25-141 and G25-40 BAC clones. **e**, **f** Structure of *WTK1* alleles from G25 (**e**) and from CS and Zavitan (**f**). Features that are unique to CS are marked with superscript CS, while those that are present only in Zavitan are marked with superscript Za

A recombinant inbred mapping population segregating for *Yr15* was generated by crossing the susceptible recurrent durum line D447 with the resistant durum *Yr15* ILs (B9, B10), into which *Yr15* had been introgressed from G25^20^. We screened 8573 F_2_ plants with DNA markers to develop a high-resolution map of the *Yr15* gene region. First, we exploited collinearity between the *Yr15* region and the syntenic regions in model organisms *Brachypodium distachyon*, *Oryza sativa* (rice), and *Sorghum bicolor* (Supplementary Fig. 4a, b and Supplementary Tables 1–4). The nearest collinearity-derived markers, *uhw264* and *uhw258*, defined *Yr15* within an interval of 0.3 cM. The target interval containing *Yr15* was then saturated with 24 markers developed from either bacterial artificial chromosome (BAC) sequences positioned on the physical map of chromosome arm 1BS of *T. aestivum* cv. Chinese Spring (CS)^24^ (Supplementary Table 5), the genome assembly of WEW accession Zavitan (Zavitan)^25^ (Supplementary Fig. 4c–e and Supplementary Table 6), or G25 BAC sequences. Using these markers, we mapped *Yr15* between markers *uhw300* and *uhw273* (0.013 cM) (Fig. 2a–c).

A pooled BAC library, constructed from *Yr15* donor G25, was screened with the closely linked or co-segregating markers *uhw297*, *uhw292*, *uhw296*, and *uhw273* (Fig. 2c), which were derived from the CS contig corresponding to the *Yr15* region. Sequencing of the six BACs isolated with these markers yielded two non-overlapping contigs (G25ctg1, 286,738 bp and G25ctg2, 131,485 bp; Fig. 2d). Sequence analysis of these contigs revealed the presence of three putative candidate genes. One of them contained predicted protein domains that previously have been associated with plant responses to pathogens and therefore was selected for validation. This gene contains two distinct kinase-like domains arranged in tandem and is designated here as *WTK1* (Fig. 2e, f). Alignment of the *WTK1* full-length complementary DNA (cDNA) against the *WTK1* genomic sequence (4657 bp) indicated that the gene contains six exons coding for a 665 amino acid protein.

### Validation of the candidate gene for *Yr15*

**Fig. 3:**
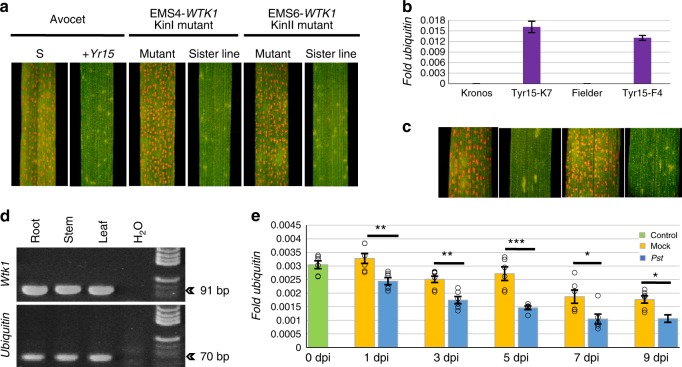
Validation of *WTK1* as *Yr15*. **a** Loss of resistance to *Pst* in *WTK1* kinase-like domain mutants. EMS4 and EMS6 carry mutations in KinI and KinII, respectively. **b**–**c** *Wtk1* IF1 transcription levels (**b**) and *Pst* resistance phenotypes (**c**) in independent transgenic events Tyr15-K2 and Tyr15-F1. Error bars denoting standard error of mean (s.e.m.) are based on three technical replicates. **d** *Wtk1* expression survey in root, stem, and leaf of B9 by RT-PCR. **e** Transcript levels of the *Wtk1* IF1 in mock-inoculated or *Pst*-inoculated B9 plants. Error bars denoting s.e.m. are based on six biological replicates. Asterisks indicate the level of significance by *t* test, *n* = 12: *p* < 0.05 (*), *p* < 0.01 (**), *p* < 0.001 (***)

To validate the function of *WTK1*, we used EMS to mutagenize a set of *Yr15* ILs and identified 2 out of 2112 tetraploid and 8 out of 1002 hexaploid M_2_ families that segregated for resistance to *Pst* (Supplementary Table 7). Sequencing of *WTK1* in the 10 susceptible M_2_ plants confirmed the presence of independent missense mutations in each mutant. Five EMS mutants contained amino acid changes in the WTK1 kinase-like domain I (KinI) and five in kinase-like domain II (KinII) (Supplementary Table 7). Examples of these mutants (EMS4, having a mutation in KinI, and EMS6, with a mutation in KinII) are presented in Fig. 3a. In the F_2_ progenies derived from crosses between the resistant wild-type Avocet + *Yr15* and two of the susceptible mutants (EMS4 and EMS6), homozygosity for the non-functional *WTK1* mutations, designated as *wtk1*, co-segregated with susceptibility to *Pst* (Supplementary Table 8). These 10 independent mutations demonstrate that both KinI and KinII domains of WTK1 are necessary for the resistance conferred by *Yr15*.

To determine whether *WTK1* is sufficient to confer resistance to *Pst*, we transformed the susceptible varieties *T. aestivum* cv. Fielder and *Triticum durum* cv. Kronos with a 9.1 kb genomic fragment harboring the complete *WTK1* coding region and its regulatory elements. Expression of the *WTK1* transgene was detected in 17 out of 22 T_0_ transgenic plants and their progenies. In subsequent generations when challenged with *Pst* (race PST-130 and isolate #5006), the *WTK1* transgene co-segregated with the yellow rust resistance phenotype (Fig. 3b, c and Supplementary Fig. 5a–g), thereby validating that *WTK1* is indeed *Yr15*.

### Expression of *Yr15* alternative transcript variants

Three alternative transcript variants—isoform 1 (IF1), isoform 2 (IF2), and isoform 3 (IF3)—were revealed by sequencing of 48 *WTK1* cDNAs (Supplementary Fig. 6a). IF1, which encodes the complete WTK1 protein, was found to be the most common transcript (85.7% of tested *WTK1* cDNAs), while the other isoforms are much less abundant (14.3% together) and encode truncated proteins. *WTK1* is expressed in roots, stems, and leaves (Fig. 3d). Transcript levels of the three *WTK1* isoforms were significantly lower in leaves of IL B9 inoculated with *Pst*, at all dpi (except 0 dpi), relative to mock-inoculated plants (Fig. 3e and Supplementary Fig. 6b). In addition, a significant reduction in expression was observed over time (1–9 dpi) for both *Pst*-inoculated (*p* *<* 8.0 × 10^−8^, *F* test, df_1_,df_2_ = 1,30) and mock-inoculated (*p* < 2.4 × 10^−5^, *F* test, df_1_,df_2_ = 1,30) plants for the *WTK1* IF1 (Fig. 3e and Supplementary Fig. 6b). These findings suggest that expression of *WTK1* is down-regulated by the presence of the pathogen.

### Changes in reading frames of non-functional alleles of *Yr15*

A search for *WTK1* sequences on chromosome arm 1BS of the CS^26^ and Zavitan^25^ whole-genome assemblies revealed only non-functional *wtk1* alleles in both of these susceptible lines (Supplementary Fig. 1a). These alleles differ from the functional *Wtk1* allele of WEW G25 by the presence of indels (e.g., insertions of transposable elements (TEs) such as *Veju*, *Harbinger*, or MITEs) that have changed the reading frame of exon 4 and generated premature stop codons in both CS and Zavitan, relative to G25 (Fig. 2e, f). In addition, orthologs of *Yr15* were found also on chromosome arms 1AS and 1DS. The predicted protein encoded by the ortholog on 1A of CS shares 83% similarity with WTK1 on 1BS of G25, but is 21 amino acids longer due to intron retentions. In contrast, the predicted proteins encoded by the 1A ortholog in Zavitan and the 1D ortholog in CS are shorter than the G25 1BS WTK1 due to frameshifts resulting in stop codons; their sequence similarities to G25 1BS WTK1 are relatively low (39.9 and 52.7%, respectively).

### Phylogeny of orthologs, paralogs, and homologs of *WTK1*

In addition to the orthologs of *WTK1* that were found on all homoeologous group 1 chromosomes of WEW (AABB) and common (AABBDD) wheats, we found *WTK1* orthologs also in diploid wheat relatives *Triticum urartu* (AA), *Aegilops speltoides* (SS), and *Aegilops tauschii* (DD), representing the ancestral A, B, and D genomes, respectively, and on chromosome 1R of rye (*Secale cereale*, RR). A phylogenetic analysis clustered all the predicted proteins into a single branch, consisting of sub-clusters corresponding to the 1A, 1B, and 1D copies (Supplementary Fig. 7). No *WTK1* ortholog was detected on chromosome 1H of barley (*Hordeum vulgare*, HH). Paralogs of *WTK1* were found on the homeologous group 6 chromosomes of tetraploid and hexaploid wheat, their diploid relatives *T. urartu* and *A. tauschii*, and on chromosome 6H of barley. All group 6 copies clustered together phylogenetically. A homologous copy from chromosome Bd2 of *B. distachyon* showed an intermediate position between the two clusters, while the closest predicted protein from *O. sativa* was resolved as an outgroup. The presence of *WTK1* paralogs on both group 1 and group 6 chromosomes of almost all diploid wheat species, as well as the relatively low nucleotide sequence identity between group 1 and group 6 paralogs of *WTK1* (~80% in KinI and ~30% in KinII), suggests an old duplication event.

### Distribution of *Wtk1* allele in wild and cultivated wheats

We have evaluated 390 *Triticum* accessions for the presence of *Wtk1* by gene-specific markers (Supplementary Tables 9–12). *Wtk1* was detected in 18% of the accessions of the southern (Israel, Lebanon, Jordan, and Syria) WEW populations but was not detected in the northern (Turkey and Iran) ones (Supplementary Table 10). We screened 93 modern durum and common wheat accessions and found that the functional *Wtk1* allele was present only in recently developed *Yr15* ILs (Supplementary Table 12).

### WTK1 TKP architecture

Both WTK1 domains belong to a protein kinase-like superfamily with a conserved catalytic domain, but contain neither membrane-targeting motifs nor known receptor sequences. The lack of transmembrane domains in WTK1 is consistent with its cytoplasmic localization (with some partition into the nucleus) in barley protoplasts transformed with a WTK1-green fluorescent protein (GFP) fusion (Supplementary Fig. 8a–i). The presence of key conserved residues^27,28^ in KinI and the lack of most of these residues in KinII implies that WTK1 possesses a putative kinase-pseudokinase structure (Supplementary Fig. 9). Moreover, KinI belongs to serine/threonine non-RD kinases, shown previously to be associated with immune receptors^29^. *WKS1* is another *Pst* resistance gene from WEW that harbors a non-RD kinase domain. However, it is attached to a lipid-binding START domain^10^ and does not exhibit high similarity to either domains of WTK1 (Supplementary Fig. 10). Only two plant resistance genes have a tandem kinase structure similar to *WTK1*: barley *RPG1*, conferring resistance to stem rust^30^, and *MLOC_38442.1*, which was proposed as a candidate for the barley true loose smut resistance gene *Un8*^31^. Nevertheless, phylogenetic analysis shows that they are quite distant from *WTK1* (Supplementary Fig. 10, 11a).

### The TKP protein family is found across the plant kingdom

**Fig. 4:**
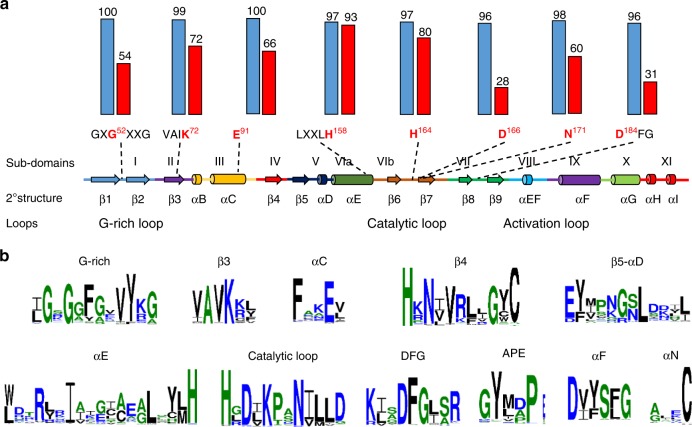
Predicted structures of putative kinase and pseudokinase domains of TKP proteins based on 92 proteins from ten plant species. **a** Conservation of key motifs, residues, and secondary structure between putative kinase and pseudokinase domains. The secondary structure of a canonical kinase is shown with the standard annotations of its subdomains^27^. Eight key residues are marked in red, with numbers that are based on their positions in α cAMP-dependent protein kinase catalytic subunit (cAPK)^27^. Dashed lines indicate positions within secondary structure elements. The histograms above the motifs represent the degree of conservation (% of identical to conserved residuals) for putative kinase (blue columns) and putative pseudokinase (red) domains. **b** Sequence logos representing the conservation of key motifs and neighboring sequences in putative kinase domains. The size of the letters corresponds to their information content

The occurrence of functional resistance genes with a tandem kinase structure (*WTK1* and *RPG1*^30^), as well as of many *WTK1* orthologs and paralogs in wheat and its near relatives, motivated us to search for similar protein architectures across the plant kingdom. Altogether, we found 92 predicted proteins that are composed of putative kinase and pseudokinase domains in tandem; like WTK1, none had additional conserved domains. Most of the putative kinase domains share key conserved residues^28^ (Fig. 4a, b and Supplementary Table 13), while the putative pseudokinase domains are generally highly divergent in these positions, suggesting that they probably have no or impaired kinase activity^32^ (Fig. 4a and Supplementary Table 14). The least conserved residues in the putative pseudokinase domains were found in the catalytic loop (D^166^ and D^184^) and the glycine-rich phosphate-binding loop (G^52^)^33^ (Fig. 4a). Analyses of motifs shared between putative kinase domains of TKP family members revealed additional conserved residues in sequences neighboring the core motifs (Fig. 4b). These results indicate that the putative kinase domains in this protein family may share a common structure.

The phylogenetic analysis showed that all 184 putative kinase and pseudokinase domains of the 92 predicted proteins could be sorted into 11 major clades and two singletons (Supplementary Fig. 11a). Homology-based comparisons of the individual kinase domains of the 92 TKPs with the annotated *Arabidopsis* kinome families^34^ revealed clear relationships between the 11 clades and specific *Arabidopsis* families or subfamilies (Supplementary Fig. 11a). Members of clades 1–10 and cluster 11.2 were found to correspond to five families of plant receptor-like kinases (RLKs): concanavalin A-like lectin protein kinases (L-LPK), leucine-rich-repeat receptor kinases (LRR), receptor-like cytoplasmic kinases (RLCK), other kinases with no published family (RK), and cell-wall-associated kinases (WAKs). Members of cluster 11.1 were not classified as RLKs. Instead, they were found to be associated with the following families of soluble kinases: cATP-dependent, cGTP-dependent, and phospholipid-dependent kinases, cyclin-dependent kinases, raf-like MAPK (mitogen-activated protein kinase) kinase kinase (MAP3K-raf), MAPK kinase kinase (MAP3K), and SNF1-related kinase 3. All 92 TKPs can be divided into two distinct groups based on the topology of the phylogenetic tree: (i) two kinase domains of the same gene that are clustered together in the same branch most likely resulted from a duplication event; (ii) two kinase domains belonging to the same gene but are positioned on separate branches represent a gene fusion event (Supplementary Fig. 11a). While four clades (2, 5, 7, and 11) included proteins that, by this criterion, resulted from either gene duplications or fusions, three clades (6, 9, and 10) included proteins that originated only from duplication events, and four clades (1, 3, 4, and 8) included proteins that resulted only from fusion events. A total of 52 TKPs were derived from duplication (occurring in kinase domains associated with 10 different families or subfamilies) and the other 40 proteins resulted from gene fusions (represented by 10 combinations of domains belonging to different families or subfamilies; Supplementary Fig. 11b). The most common domains present in TKPs were similar to WAK kinases (63 out of 184) and LRR_8B (cysteine-rich kinases; 49 out of 184). WTK1 appears to be derived from a fusion of WAK and RLCK_8 kinase domains. Interestingly, most fusion events were found in monocot TKPs. Notably, clade 6 includes proteins of distant species and was presumably originated from a single ancient duplication event of a kinase in the L-LPK family before the divergence of the monocots and eudicots.

## Discussion

Although new *R*-genes are frequently discovered from various sources, the rapid evolution of *Pst* populations overcome most of the commonly used *Yr* genes, rendering most of the world wheat production susceptible to devastating epidemics^4^. The combination of marker-assisted selection and classical approaches can speed up the progressive incorporation of individual new *R*-genes into elite wheat varieties. However, it seems that the appearance of new virulent *Pst* races in the fields is faster than the incorporation of novel *R*-genes into elite wheat varieties.

*Yr15* and *Yr5* showed broad-spectrum resistance to *Pst* races from around the globe^8^ and are among the most promising genes for protecting wheat yields. Nevertheless, virulent races were detected against each one of them, separately, in Australia for *Yr5*^22^ (isolate AU85569) and in Europe for *Yr15*^19^ (e.g., isolate DK92/02). Therefore, we were interested to know whether the pyramiding of *Yr5* and *Yr15* into one cultivar could provide a more durable resistance. Our results indeed show that *Yr5* and *Yr15* complement each other and provide full resistance to the virulent races. Pyramiding resistance genes by classical breeding methods may take many years. However, approaches based on genetic transformation of individual cloned *R*-genes or of R-cassettes that include several *R*-genes with complementary resistances to various races, preferably via different mechanisms, can be considerably faster and can also overcome the problem of individual genes segregating in backcross populations. *Yr5*, *Yr15*, *Yr18*, *Yr36*, and *Yr46* are obvious candidates to be included in such a cassette, since they represent different gene architectures (NLR, kinase-pseudokinase, ABC transporter, Kinase-START, and hexose transporter, respectively^7^) and most likely confer resistance via different molecular mechanisms.

The comparison of fungal development in susceptible vs. resistant NILs presented here provides indications for the possible mechanism of resistance conferred by *Yr15*. The macroscopic and microscopic observations, together with the quantification of fungal biomass in the resistant genotype that carries *Yr15*, show clear development of HR after fungal penetration, establishment of fungal feeding structures within the plant cells and restriction of fungal growth. The microscopic observations showed that a strong PCD response took place as part of the resistance mechanism, resulting in localized cell death that arrested further colonization of host cells by *Pst*^5,6^. We showed that the size of chlorotic spots as a macroscopic indication of HR varied between the different genetic backgrounds of the *Yr15* ILs. The HR/PCD response can be activated via different cellular pathways;^5^ further experiments are needed to identify the proteins interacting with WTK1 and the exact pathway that activates HR, in order to provide a better understanding of the mechanism of action and the host–parasite co-evolutionary processes behind it.

Two out of the six *Yr* genes cloned in wheat are derived from WEW (*Yr36* (*WKS1*) and *Yr15* (*Wtk1*)) and provide broad-spectrum resistance against *Pst*. Both are present only in the southern distribution range of WEW populations in the Fertile Crescent (Israel, Lebanon, Jordan, and Syria) and were not detected in the northern populations (Turkey and Iran). Since wheat was probably domesticated from wild emmer in southeast Turkey^1^, both *Yr15* and *Yr36* have, until recently, not found their way into the genepool of cultivated wheat germplasm. While the *WKS1* sequence is completely absent from all three genomes of hexaploid wheat^10^, in the current study we have shown that non-functional orthologs and paralogs of WTK1 are present on all chromosomes of groups 1 and 6 of the tetraploid (AABB) and hexaploid (AABBDD) wheat genomes. Despite the relatively low similarity of orthologous and paralogous copies of WTK1 proteins in wheat that are associated with the susceptibility phenotype (Zavitan and CS), they still may carry resistance to some pathogens in other genetic backgrounds (such as diploid species) or may have other functional alleles in natural WEW populations. Furthermore, the presence of orthologs, paralogs, and homologs of WTK1 in all ancestral diploid genomes of wheat, and even in the basal Pooid *Brachypodium*, indicates that WAK-RLCK kinase-pseudokinase fusion represents an old event that occurred prior to the divergence of the wheat and *Brachypodium* lineages, about 35–40 million years ago^35^. In contrast, the kinase-START protein architecture of WKS1 has not been found in other species outside of the Triticeae tribe and is likely a recent fusion of existing kinase and START domains^10^. For current breeding purposes, both *Yr15* and *Yr36* constitute untapped resources to improve resistance to *Pst* in domesticated wheat, since they are absent in all tested modern durum and common wheat varieties, except for recent introgressions.

The kinase protein superfamily represents 1–2% of the functional genes across plant and animal genomes^36^, as reflected by the wide involvement of kinases in almost all cellular processes. The RLK clade is relatively expanded in flowering plants^36^ and plays an important role in plant immune responses^37^. The current phylogenetic analysis of the TKP protein family revealed that 175 out of 184 kinase domains were associated with RLKs, suggesting that TKPs are involved in plant defense mechanisms. Moreover, broad-spectrum resistance to biotrophic fungal pathogens (e.g., rusts and smut) was already demonstrated for *WTK1* and *RPG1*^30^ and proposed for *Un8*^31^, raising the question of whether the widespread TKPs serve generally as a family of resistance proteins. Most RLKs in *Arabidopsis* share multi-domain architecture with receptor, transmembrane, and kinase domains^38^, but 24% of them contain no extracellular or transmembrane domains, which are likewise lacking from TKP family members. Moreover, both RPG1^30^ and WTK1 are localized mainly in the cytoplasm.

The evolution of multi-domain proteins, such as TKPs, presumes monophyletic or polyphyletic origins, respectively, from single or multiple independent evolutionary events^39^ that can be explained by single or multiple birth models for protein families^40^. Our phylogenetic analysis indicates that TKP family members originated from either gene duplication or gene fusion, suggesting a polyphyletic origin of the TKPs. Moreover, even divergent members of the TKP family, such as RPG1^30^ and WTK1, which evolved, respectively, by duplication and fusion, share not only similar gene architecture but also analogous function (e.g., resistance to pathogens). Most of the TKP proteins (52 out of the considered 92) were formed by tandem duplication, earlier shown to be more frequent than fusion during the evolution of multi-domain proteins^39^. In addition, we have seen indications on lineage-specific origin of some TKP members, that is, domains related to WAK family that were found only in monocots.

After gene duplication or fusion, one of the copies can gain a new function, a process known as neofunctionalization^41^. Interestingly, all members of the TKP family discussed here possess putative kinase-pseudokinase domain architecture and probably fit this neofunctionalization model. This structure may be related to the function of TKPs since some pseudokinases were shown to play an important role as baits for pathogen effectors^42^. Moreover, 20% of the RLK family members are pseudokinases, a higher percentage than the average in the *Arabidopsis* kinome (13%)^36^. The WTK1 pseudokinase belongs to the RCLK family. Members of this family were shown to serve as targets for inhibition by pathogen effectors (e.g., BIK1 in *Arabidopsis*)^43^. Moreover, the duplication event itself was suggested as a source for the decoy model^44^. Thus, the decoy role can be proposed as one of the potential mechanisms of function of the TKP family members in immune response. However, further studies are required in phylogenetically representative plant species in order to elucidate the mechanism of resistance conferred by this unique protein family.

Furthermore, members of the animal Janus kinase (JAK) family contain a similar structure of kinase and pseudokinase domains, a unique feature of JAKs compared to other animal protein kinases^45^. The participation of members of both JAK^46^ and TKP (e.g., WTK1) families in innate immune responses, particularly in PCD, calls attention to not only structural but also possibly functional similarities between these proteins, suggesting convergent molecular evolution of proteins involved in immunity in both the plant and the animal kingdoms.

## Methods

### Mapping populations and yellow rust assays

The yellow rust resistance gene *Yr15* was introgressed from the resistant WEW accession G25 (G25) into the susceptible durum wheat (*T. turgidum* ssp. *durum*) accession D447 (LD393/2*Langdon ND58-322) to develop the resistant BC_3_F_9_ (B9) or BC_3_F_10_ (B10) ILs^21^. A large mapping population, consisting of 8573 F_2_ plants, was developed by crossing D447 with these ILs (B9 or B10), which carry *Yr15* within a segment of chromosome arm 1BS from G25.

Phenotyping of recombinant, mutagenized, and transgenic lines under growth chamber conditions was carried out with Israeli *Pst* isolate #5006 (race 38E134) and US *Pst* race PST-130 using a standard protocol^21^. Plants were inoculated either at the two-leaf to four-leaf stage (“seedling inoculation”) or at the stem elongation stage (“adult-plant inoculation”). The yellow rust response variation was evaluated 14 to 18 dpi using a 0 to 9 scale of IT^47^.

*Yr15* and *Yr5* ILs were phenotyped for their response to *Pst* isolates DK92/02 and AU85569 from the isolate collection of the GRRC (Aarhus University, Flakkebjerg, Slagelse, Denmark). Plants were point inoculated with urediniospores suspended in engineered fluid (NovecTM 7100; 3M, Maplewood, MN, USA)^48^. Using a 0 to 9 scale^47^, the yellow rust responses were scored 18 dpi; the phenotypes were recorded using a Canon EOS 7D digital SLR camera equipped with a Canon macro EF 100mmf/2.8L IS USM lens (Canon, Tokyo, Japan).

### Microscopy of *Pst*–wheat interactions within infected leaves

Fluorescence microscopy of *P. striiformis* structures was performed using wheat germ agglutinin (WGA; a lectin that binds specifically to β (1→4)-*N*-acetyl-d-glucosamine, i.e., chitin) conjugated with a fluorescent dye^49^. Leaf segments (second leaf, 10 cm long) from the NILs, Avocet S, and Avocet + *Yr15*, each of which had been inoculated with urediniospores of *Pst* isolate #5006, were sampled from 1 until 11 dpi (10:00 to 10:30 a.m., every 2 days), when visible sporulation developed on the susceptible plants. Fluorescence microscopy was carried out on an inverted fluorescence microscope, Leica DMi8 (Leica Microsystems, Wetzlar, Germany), fitted with a filter cube for the FITC excitation range (Ex: 460–500; Dc: 505; Em: 512–542), and a FLUO regime to observe the WGA-stained fungal structures.

### Quantification of fungal biomass within infected leaves

After inoculation with *Pst* isolate #5006, leaf segments (middle, second leaf) of the resistant Avocet + *Yr15* and susceptible Avocet S NILs were cut every 2 days (1–14 dpi) from different plants at each time point, with eight biological replicates collected for each line at each time point, and used for quantification of fungal biomass by chitin measurement^50^. Three technical replicates were made for each tissue sample. Fluorescence was measured on a SpectraMax M2e Microplate Reader (Molecular Devices, Sunnyvale, CA, USA), using 485-nm excitation and 535-nm emission wavelengths, a 1.0 s measurement time, and a cross pattern of well-scanning, yielding an average measurement per well. Statistical analyses of parameters associated with the accumulation of fungal biomass were determined by the maximum likelihood estimation method (SPSS) following the logistic population growth model. The null hypothesis (no growth) was tested by the likelihood ratio test.

### Confocal microscopy

The second leaf of 16-day-old plants of the susceptible Avocet S and resistant Avocet + *Yr15* NILs were point inoculated^48^ with fresh spores of the *Pst* isolate UK75/30. One leaf segment per plant was sampled at 4 and 8 dpi. Leaf samples were fixed in ethanol:chloroform (3:1, v/v) + 0.15% (w/v) trichloroacetic acid solution for at least 24 h. Segments were then washed twice in 50% ethanol (10 min) followed by clearing in 0.05 M NaOH (30 min). After washing with deionized water (DI) and 0.1 M Tris-HCl buffer (pH 5.8) (30 min), samples were stained for 10 min in Tris-HCl buffer containing 0.1% Uvitex 2B (Polysciences Inc.) (w/v). Specimens were washed four times in DI followed by one wash in 25% glycerol and were left overnight in DI to remove access stain. Specimens were stored in 50% glycerol until further use.

For the microscopic investigations, leaf segments were mounted on glass slides in 75% glycerol. Fungal colonies and host cell autofluorescence were analyzed using an Olympus FV1200 confocal laser scanning microscope. Fungal structures and leaf tissues were excited with 405 and 515 nm lasers, and the emitted light was detected in two channels with filter settings 486–520 and 554–654 nm, respectively. Z-stacks were collected with 1 µm separation. The images from the two detection channels were merged and 3D projections were performed with IMARIS^®^ bitplane 6.2. The resulting 2D images were adjusted for brightness and contrast. This included small adjustments in individual color channels to obtain an equal representation of the background signal from the healthy plant tissue on all images. All adjustments were applied equally across a whole image using the free software paint.net (https://www.getpaint.net/).

### Development of a high-density genetic map

We assigned *Yr15* to deletion bin Sat0.31 of chromosome arm 1BS by developing and mapping dominant marker *uhw250*, as well as cleaved amplified polymorphic sequences^51^ markers *uhw252* and *uhw254*, based on expressed sequence tags assigned to Sat0.31^52^ (Supplementary Table 2). Using the GenomeZipper approach^24,51^, which infers collinearity with orthologous regions of *B. distachyon*, *O. sativa*, and *S. bicolor* (Supplementary Table 3), eight additional markers were developed and the *Yr15*-containing region was reduced to a 0.3 cM interval between flanking markers *uhw264* and *uhw259* (Supplementary Fig. 4a).

Single-nucleotide polymorphism (SNP) markers *RAC875_c826_839* and *BS00022902_51* derived from the 15K wheat SNP array (Trait Genetics GmbH, Gatersleben, Germany) were converted to Kompetitive Allele-Specific PCR (KASP) markers using PolyMarker (http://polymarker.tgac.ac.uk/) (Supplementary Table 4).

The screening of F_2_ plants from the D447 × B9 and D447 × B10 crosses for recombination events in the region carrying the *Yr15* gene was carried out with the following marker sets (Supplementary Fig. 4b): (i) *wmc406* and *gwm273* (*6*); (ii) *RAC875_c826_839* and *BS00022902_51*; (iii) *uhw264* and *uhw259*. A total of 94 homozygous recombinant inbred lines (RILs), corresponding to 13 independent recombination events, were detected and used for the high-density map and for chromosome walking. These RILs were evaluated for resistance to *Pst* isolate #5006.

### Physical maps of the *Yr15* region on chromosome arm 1BS

Markers *uhw264* and *uhw259*, flanking *Yr15*, were used to screen a gridded CS BAC library^53^. Three positive BAC clones (TaaCsp364O11, TaaCsp1023G2, and TaaCsp1158K20) were detected with marker *uhw264* and two (TaaCsp729H14 and TaaCsp814G12) with *uhw259* (Supplementary Fig. 4b-d). The BAC-end sequence (BES) of clone TaaCsp364O11 was used to develop marker *uhw267*. Screening of the CS library with *uhw267* yielded clone TaaCsp691F7. The BES of clone TaaCsp729H14 was used to develop marker *uhw268*, which was mapped distal to *uhw259* (Supplementary Fig. 4c). These efforts generated two BAC contigs, flanking *Yr15*, with a gap between them, which was bridged using a 1BS physical map constructed from a CS 1BS-specific BAC library^24^. The contigs of the CS 1BS physical map that cover the region corresponding to *Yr15* were assembled as follows^24^. A wheat BAC library composed of 55,296 BAC clones was constructed from CS 1BS. In total, 49,412 high-quality fingerprints, representing 14.4 1BS equivalents, were assembled into 57 long scaffolds covering 83% of chromosome 1BS using the LTC software^24^. The LTC network representation of significant clone overlaps of the scaffold covering the *Yr15* region is presented in Supplementary Fig. 4e. Screening of the 57 three-dimensionally arrayed BAC pools that comprise the minimal tiling path (6447 BACs selected from 49,412 fingerprinted clones) with markers *uhw264*, *uhw267*, *uhw268*, and *uhw259* identified a 1.3 Mb contig spanning the complete *Yr15* region (ctg49; Supplementary Fig. 4e). The 21 BAC clones (Supplementary Fig. 4d) that bridged the gap between markers *uhw267* and *uhw268* were identified and sequenced. Molecular markers developed from the sequenced clones of the 1BS contigs yielded genetic markers *uhw297* and *uhw292*, which are distal to *Yr15*, *uhw296*, and *uhw276*, which co-segregate with *Yr15*, and *uhw273*, *uhw275*, *uhw291*, *uhw274*, *uhw282*, *uhw284*, and *uhw277*, as well as physical markers *uhw280* and *uhw279*, which are proximal to *Yr15* (Supplementary Table 5 and Supplementary Fig. 4c). The names and order of the 21 BAC clones are presented in Supplementary Fig. 4d.

The *Yr15* donor line, G25, was used for construction of a pooled BAC library^54^. High molecular weight genomic DNA was partially digested with *Hind*III to obtain fragments with sizes in the range of 100–250 kb, which were ligated into a pINDIGO vector (Caltech, Pasadena, CA, USA) and transformed into *Escherichia coli* cells^54^. After growing individual *E. coli* colonies on agar plates, 443,880 transformed colonies were collected into 150 pools having an average of 2959 colonies per pool. The genome coverage of the G25 BAC library was calculated as 4.5× (average clone size, 120 kb). The initial screening of the library with marker *uhw280* yielded BAC clone G25-64, which was sequenced and used to develop the proximal markers *uhw288*, *uhw289*, *uhw287*, *uhw285*, *uhw286*, and *uhw281* (Supplementary Table 5). Further screening of the G25 BAC library with the closest distal markers *uhw297* and *uhw292*, the co-segregating marker *uhw296*, and the proximal marker *uhw273* yielded six BAC clones (G25-33, G25-86, G25-40, G25-141, G25-35, and G25-25; Fig. 2). DNA samples of these BACs were extracted using a Qiagen Plasmid Midi Kit or Qiagen Large-Construct Kit (Qiagen, Hilden, Germany). Contiguous sequences were generated on a Pacbio RS II (Pacific Biosciences, Menlo Park, CA, USA) at the Institute of Biotechnology, University of Helsinki (Helsinki, Finland). BAC clones were sequenced and assembled (HGAP3 implemented in SMRT portal 2.3) separately. The GAP4 program (Staden package) was used to edit and join the assembled BAC contigs into two contigs (G25ctg1, 286,738 bp; and G25ctg2, 131,485 bp) spanning the *Yr15* gene region (Fig. 2). One *Yr15* distal marker (*uhw300*) and two co-segregating markers (*uhw302* and *uhw301*) were developed by comparing of G25 sequences to the 1BS pseudomolecule of Zavitan^25^ (Supplementary Table 6).

### G25 contig annotation and identification of candidate genes

Repetitive elements were masked using the Triticeae Repeat Sequence Database (TREP; http://botserv2.uzh.ch/kelldata/trep-db/index.html). Non-repetitive sequences were analyzed for genes by BLASTN searches against the GenBank (https://blast.ncbi.nlm.nih.gov/Blast.cgi) and the TIGR Wheat Genome (http://tigrblast.tigr.org/euk-blast/index.cgi?project=tae1) databases, using Genscan (http://genes.mit.edu/GENSCAN.html) and FGENESH (http://www.softberry.com/berry.phtml). A BLASTN search of the sequences of the G25ctg1 and G25ctg2 contigs against the high confidence (HC) gene models of the 1BS pseudomolecule of Zavitan^25^ was used to reveal the presence of putative HC candidate genes.

### Isolation and sequencing of the full-length *WTK1* cDNA

Total RNA was extracted with the TRIzol reagent (Invitrogen, ThermoFisher Scientific, Waltham, MA, USA). Poly(A)+ RNA was purified from the total RNA with a Qligotex mRNA Midi Kit (Qiagen). First-strand cDNA was synthesized with Superscript II (Invitrogen) using primer E1820 (Supplementary Table 9). Nested PCR was carried out first with E1820 and the *Yr15* 5′-untranslated region (UTR) primer Y15F0 and then with E2146 (matching part of E1820; Supplementary Table 9) and the 5′-UTR primer Y15F2. The PCR products were purified, cloned, and sequenced.

To determine the start site of the *WTK1* transcript, we used 5′ rapid amplification of cDNA ends (RACE)^55^. In brief, the poly (A)+ RNA was treated with tobacco acid pyrophosphatase (Epicentre, Madison, WI, USA), and then purified with an RNeasy Plant Mini Kit (Qiagen). An adapter (RNAoligo, Supplementary Table 9) was ligated to the 5′ end of the RNA and reverse transcription was carried out as above, primed with E1820. Amplification of the 5′ end was carried out by two rounds of PCR reactions, using the primer 5′ RACE (Supplementary Table 9) and sequentially two gene-specific primers, Y15R2 and Y15R1 (Supplementary Table 9).

### Candidate gene validation by screening EMS-mutagenized lines

Seeds of the *Yr15* durum wheat (B9) and common wheat (Avocet + *Yr15*, Excalibur + *Yr15*, and Suncea + *Yr15*) ILs were treated with 0.4–0.75% EMS solution for 16–18 h at room temperature^56^. Prior to sowing, the seeds were washed three times with 10% sodium thiosulfate and then twice in water (30 min each time), covered with Whatman paper, and air-dried at 4 °C.

EMS-treated M_1_ plants were generated at the University of Haifa (Haifa, Israel) and at the University of Sydney (Cobbitty, Australia). M_2_ families (10–20 seeds per family) were artificially infected with *Pst* under field conditions in Israel (*Pst* isolate #5006), or under greenhouse conditions in Australia (*Pst* isolate 110 E143A+). All M_3_ seedlings obtained from susceptible M_2_ plants were inoculated in a growth chamber with *Pst* isolate #5006 to confirm the homozygosity of the recessive mutations, and then screened for mutations within the coding sequence of the *Yr15* candidate gene *WTK1*. PCR products were amplified from genomic DNA with gene-specific primers (Supplementary Table 9), sequenced, and then compared for nucleotide variations by multiple sequence alignment. The full-length cDNA of *WTK1* was amplified from the susceptible M_2_ plants and sequenced using two cDNA-specific primer pairs (*WTK1_L2F* and *WTK_RE6*, Supplementary Table 9), in order to confirm the detected point mutations at the messenger RNA (mRNA) level. The *WTK1* mutations were ranked with SIFT (http://sift.jcvi.org/) to predict the effects of non-synonymous mutations on protein function (Supplementary Table 7).

We crossed two of the EMS-mutagenized lines of common wheat (EMS4 and EMS6, Supplementary Table 7) with the resistant wild-type parental line Avocet + *Yr15* to produce segregating F_2_ families. The response of each F_2_ population to yellow rust was assessed at the seedling stage; goodness of fit for the observed and expected ratios in that population was evaluated with a *χ*^2^ test (Supplementary Table 8). Sequence analysis of PCR products amplified from 40 F_2_ plants was carried out as described above to confirm co-segregation of homozygosity for the mutations in *WTK1* with the loss of resistance to *Pst*.

### Candidate gene validation by transgenic complementation

*Agrobacterium tumefaciens*-mediated transformation of susceptible durum and common wheat varieties, respectively Kronos and Fielder, served to further verify *WTK1* function by complementation. Phusion^®^ High-Fidelity DNA Polymerase (New England BioLabs, Ipswich, MA, USA) was used to amplify the *WTK1* genomic region from G25 BAC clone G25-141 (Fig. 2). Restriction sites *Xho*I and *Avr*II were added, respectively, to primers *Yr15F1/R1* and *Yr15F2/R2* (Supplementary Table 9) to enable cloning into the pLC41Hm transformation vector. Two overlapping PCR products were digested by the restriction enzyme pairs *Xho*I–*Msc*I and *Msc*I–*Avr*II, purified from agarose gel bands, and cloned into *Xho*I–*Spe*I linearized pLC41Hm. A 9116-bp genomic fragment that contains the full-length *Wtk1* coding region, as well as 3428 bp upstream of the start codon and 1031 bp downstream of the stop codon, was cloned for transformation. Sanger sequencing confirmed the accuracy of the construct. *Agrobacterium tumefaciens*-mediated transformation^57^ of Kronos and Fielder with this construct was carried out at the University of California Plant Transformation Facility (Davis, CA, USA) (http://ucdptf.ucdavis.edu/).

In total, 15 independent T_0_ plants in Kronos (Tyr15-K1 to Tyr15-K15) and 7 T_0_ plants in Fielder (Tyr15-F1 to Tyr15-F7) were obtained. Three primer pairs (*HpyF1/R1*, *Yr15TestF1/R1*, and *Y15K1_F2/Yr15P2*; Supplementary Table 9) served to validate the presence of *WTK1* in the transgenic plants. In addition, we extracted mRNA from all T_0_ plants and estimated the transcript levels of the three *Wtk1* isoforms (isoform 1, IF1; isoform 2, IF2; isoform 3, IF3) by quantitative real-time PCR (qRT-PCR) using *Ubiquitin* as the endogenous control.

We germinated 10–25 T_1_ seeds from each transgenic event that expressed *WTK1* and inoculated the plants with *Pst* race PST-130, which is virulent on Kronos and Fielder. All 12 positive T_1_ families in Kronos and the 5 positive T_1_ families in Fielder showed resistance to this *Pst* race. We genotyped all tested T_1_ plants and confirmed co-segregation of the resistance with the presence of the transgene. Furthermore, 5 to 10 independent plants each of the T_2_ families from transgenic events Tyr15-F1, Tyr15-F4, Tyr15-F5, Tyr15-F6, Tyr15-K7, Tyr15-K8, Tyr15-K10, Tyr15-K12, and Tyr15-K15 were also tested with *Pst* isolate #5006, which is likewise virulent on Kronos and Fielder.

### Gene expression analysis by quantitative RT-PCR

Expression analysis was conducted with total RNA isolated from various plant tissues (leaves, roots, and stems) using an RNeasy Plant Mini Kit (Qiagen). First-strand cDNA was generated using the qScript TM Flex cDNA Synthesis Kit (Quanta Biosciences, Beverly, MA, USA). The qRT-PCR was performed on a StepOne Plus Real-Time PCR System (Applied Biosystems, Foster City, CA, USA) using the following program: 95 °C for 20 s; 40 cycles of 95 °C for 3 s and 60 °C for 30 s. The qRT-PCR reaction mixture contained the following components in a total volume of 10 µl: 5 µl Fast SYBR green master mix (Applied Biosystems); 2.5 µl diluted cDNA; 300 nM of each primer. The efficiency of each pair of primers was calculated using four serial fivefold dilutions (1:1, 1:5, 1:25, and 1:125) in triplicates. Amplification efficiencies of all primers were higher than 95%. Transcript levels are expressed as linearized fold-*Ubiquitin* levels calculated by the formula 2^(Ubiquitin CT−Target CT)^ ± standard error of the mean (SEM).

### Assessment of alternative splicing of *WTK1* mRNA

Alternative splicing variants (IF1, IF2, IF3; Supplementary Fig. 6a) were revealed by sequencing of 48 B9 cDNA clones, which were amplified with a poly(T) primer and *WTK1-*specific primers, or with *WTK1*-specific primers alone. To determine the relative transcript levels of these three splicing variants, we designed PCR primers for each *WTK1* isoform (Supplementary Table 9). Leaves of B9 were collected after the following treatments: (i) before inoculation (0 h), as the control; (ii) after inoculation with spores of *Pst* isolate #5006 suspended in Soltrol^®^ 170 light oil; (iii) after spraying with Soltrol^®^ 170 light oil lacking *Pst* spores, as a mock control. Samples of treatments (ii) and (iii) were collected at 1, 3, 5, 7, and 9 dpi, with six biological replicates for each treatment at each time point. All data were subjected to statistical analysis using the general linear model in SPSS.

### Structure of *wtk1* from CS and Zavitan

A search for *WTK1* sequences in the chromosome arm 1BS of CS^26^ and in the Zavitan^25^ whole-genome assemblies revealed the presence of non-functional alleles in both of these susceptible lines. The presence of three TEs: (i) a non-autonomous TRIM LTR retrotransposon of the Veju family (RLX_Taes_Veju) in intron 1 of CS and Zavitan); (ii) a DNA transposon of the DTH_Harbinger family (trep3042) in intron 3 of CS and Zavitan; (iii) a non-autonomous DNA transposon of the DTM_MITE type (trep1674-1) in intron 3 of CS, was identified by a search in two TE databases—mipsREdat_9.3p_Poaceae_TEs and trep-db_complete_Rel-16.

### Evolutionary history of cereal WTK1 orthologs and paralogs

WTK1 protein sequences, used for phylogenetic analyses, were obtained from the genome assemblies of WEW (Zavitan pseudomolecules)^25^, common wheat (CS pseudomolecules)^26^, barley *H. vulgare* (Morex pseudomolecules) (http://webblast.ipk-gatersleben.de/barley_ibsc/), *A. speltoides* (accession #29; https://wheat-urgi.versailles.inra.fr/Seq-Repository/Assemblies), *A. tauschii* (http://plants.ensembl.org/Aegilops_tauschii/), *T. urartu* (NCBI number PRJNA182347), and rye *Secale cereale* (http://webblast.ipk-gatersleben.de/ryeselect/).

The public resource eggNOG version 4.5 (http://eggnogdb.embl.de) was searched for WTK1 Orthologous Group proteins at various taxonomic levels. The resulting set of WTK1 orthologs, designated as ENOG4115QHQ, included 11 protein sequences from nine different species. Two of these proteins were used for further phylogenetic analysis (*B. distachyon* BRADI2G38370.1 and *O. sativa* LOC_Os01g20880.1).

The analysis included 21 protein sequences. All sequence positions that contained gaps, as compared with WTK1-1B from G25, were eliminated from the analysis. Therefore, the analysis was conducted using 263 amino acid residues out of the 665 of the full-length WTK1 predicted from the DNA sequence of clone G25-141. The evolutionary history of WTK1 was inferred using the neighbor-joining algorithm^58^ based on evolutionary distances computed by the Poisson correction method^59^. The quality of the derived phylogeny of WTK1 was assayed using a bootstrap test with 10,000 replicates^60^. The analysis was performed with MEGA7 (https://www.megasoftware.net/).

### Distribution of *WTK1* among various *Triticeae* species

A set of two gene-specific, diagnostic markers, one for KinI and one for KinII (Supplementary Table 9), was developed and then used to test the distribution of *Wtk1* among various *Triticeae* species. These dominant PCR markers were designed to identify the presence of the *Wtk1* functional allele, amplifying a 1 kb PCR product for KinI and 2 kb for KinII.

### Subcellular localization of the WTK1 protein

Barley protoplasts (8 × 10^5^) were isolated^61^ from *H. vulgare* cv. Bomi plants and electroporated with 20–40 µg DNA at 300 V/cm essentially as described before^61^, diluted into Gamborg’s B-5 Basal Medium with minimal organics that contained 10% glucose, and then transferred into glass-bottomed 35 mm microwell dishes (MatTek Corporation, Ashland, MA, USA) for microscopy.

Electroporated protoplasts were cultured overnight at 22 °C before imaging under an inverted confocal laser scanning microscope (Leica TCS SP5 II; Leica Microsystems, Wetzlar, Germany) with a ×63 water immersion objective. The following light ranges were used: GFP (Ex 488 nm, Em 500–543 nm); 4′,6-diamidino-2-phenylindole (Ex 405 nm, Em 430–550 nm); chlorophyll autofluorescence (Ex 488 nm, Em 673–725 nm).

A *WTK1* cDNA clone was prepared as described under Isolation and sequencing of full-length *WTK1* cDNA. All expression vectors were created with the Multisite Gateway Three-Fragment Vector Construction Kit (Invitrogen). The 5′ entry clone was prepared by the BP reaction between pDONRP4-P1R and a 35S promoter fragment amplified from pBI221 (GenBank accession #AF502128.1) using primers attB4F35S and attB1R35S (Supplementary Table 9). The entry clone for the full-length *WTK1* was created by carrying out the BP reaction between pDONR221 and the PCR product amplified from the *WTK1* cDNA clone with primers attB1FKinase and attB2RYr15 (Supplementary Table 9). The entry clone for the N-terminal and C-terminal kinase-like domains were similarly prepared using primers attB1FKinase and attB2Rkinase, and attB1Freg and attB2RYr15 (Supplementary Table 9), respectively. The 3′ entry clone was prepared by carrying out the BP reaction with pDONRP2RP3 and a GFPnos fragment amplified from pVEC8_GFP (GenBank: FJ949107.1) by PCR using primers attB2FGFPnos and attB3RGFPnos (Supplementary Table 9). The final clones used for transient transformation and expression in protoplasts were created by the LR reaction between the respective entry clones and the destination vector pDESTR4-R3 according to the instructions of the Gateway Kit.

### Analysis of WTK1 kinase-like domains

A BLASTP search of the NCBI non-redundant protein database was used to assign the WTK1 kinase-like domains to a specific kinase superfamily and to search for proteins similar to WTK1^62^. Multiple alignments of WTK1 kinase domains with 23 different plant kinase domains were performed using Clustal Omega with default parameters^63^. In this analysis, we included the 4 closest kinases identified by BLASTP (WKS1, PTO, and two of WAK5), the 2 kinase domains of RPG1^30^, the 2 kinase domains of *H. vulgare* MLOC_38442.1^31^, and 15 kinase domains from known and putative plant pattern recognition receptors, of which 14 are non-RD kinases and 1 is an RD kinase^29^ (Supplementary Fig. 9). A phylogenetic tree was computed with RaxML^64^ and drawn with iTOL (https://itol.embl.de/) to visualize the relationships between the different groups of kinases (Supplementary Fig. 10).

### Analysis of the TKP family in plants

A search for predicted proteins with a TKP structure was conducted in whole-genome assemblies of various plant taxa: *O. sativa* (http://rapdb.dna.affrc.go.jp/download/irgsp1.html), *Zea mays* (http://ensembl.gramene.org/Zea_mays/), *H. vulgare* (http://webblast.ipk-gatersleben.de/barley_ibsc/), *Secale cereale* (http://webblast.ipk-gatersleben.de/ryeselect/), *T. aestivum*^14^, *S. bicolor* (https://phytozome.jgi.doe.gov/pz/portal.html#!info?alias=Org_Sbicolor), *Arabidopsis thaliana* (https://www.arabidopsis.org/download/index-auto.jsp?dir=/download_files/Proteins), *Physcomitrella patens* (https://plants.ensembl.org/Physcomitrella_patens/), *Solanum tuberosum* (http://plants.ensembl.org/Solanum_tuberosum/), *Brassica napus* (http://www.genoscope.cns.fr/brassicanapus/data/), and *Populus trichocarpa* (https://phytozome.jgi.doe.gov/pz/portal.html#!info?alias=Org_Ptrichocarpa). Kinomes were extracted according to functional annotations of the gene models of the above genome assemblies. Conserved kinase domains were identified using ProSITE (https://prosite.expasy.org/) and CD-Search (https://www.ncbi.nlm.nih.gov/Structure/cdd/wrpsb.cgi), the predicted proteins with putative tandem kinases were extracted for further analysis. Predicted tandem kinase proteins with incomplete kinase domains were excluded from further analysis.

In total, 89 predicted protein sequences were selected for further analyses: *T. aestivum* (38 proteins), *H. vulgare* (8), *Secale cereale* (3), *O. sativa* (10), *Z. mays* (8), *S. bicolor* (12), *A. thaliana* (1), *Physcomitrella patens* (1), *Solanum tuberosum* (1), *Brassica napus* (5), and *P. trichocarpa* (2). In addition, the sequences of WTK1 from G25, RPG1^30^, and MLOC_38442.1^31^ were included in the analysis to represent the tandem kinase structure, for which a function was assigned or proposed. Predicted protein sequences were aligned with MUSCLE (https://www.ebi.ac.uk/Tools/msa/muscle/). The evolutionary relationships between predicted proteins having a tandem kinase structure was inferred by the maximum likelihood method based on the JTT matrix-based model^65^. The bootstrap consensus tree was calculated from 1000 replications^60^. Branches were merged when a node was found in <50% of the bootstrap replicates (Supplementary Fig. 11a). The analysis was performed with MEGA7 (https://www.megasoftware.net/).

Graphical representation of amino acid conservation based on sequence logos was performed using WebLogo 3 (http://weblogo.threeplusone.com/). The presence of key conserved residues^27^ was used to predict the catalytic activity of each domain.

## Electronic supplementary material

Supplementary Information

## Data Availability

Sequences have been deposited in GenBank under accession numbers MG649384 and MG674157. The authors declare that all other data supporting the findings of this study are available within the manuscript and its supplementary files or are available from the corresponding author upon request.
