# Supplementary material for: Cloning of the wheat Yr15 resistance gene sheds light on the plant tandem kinase-pseudokinase family
Source: Nat Commun. 2018 Oct 3;9:3735. doi: 10.1038/s41467-018-06138-9 (PMC6170490; doi:10.1038/s41467-018-06138-9)
Supplement: Supplementary file 1 — Supplementary Information [file 41467_2018_6138_MOESM1_ESM.pdf]

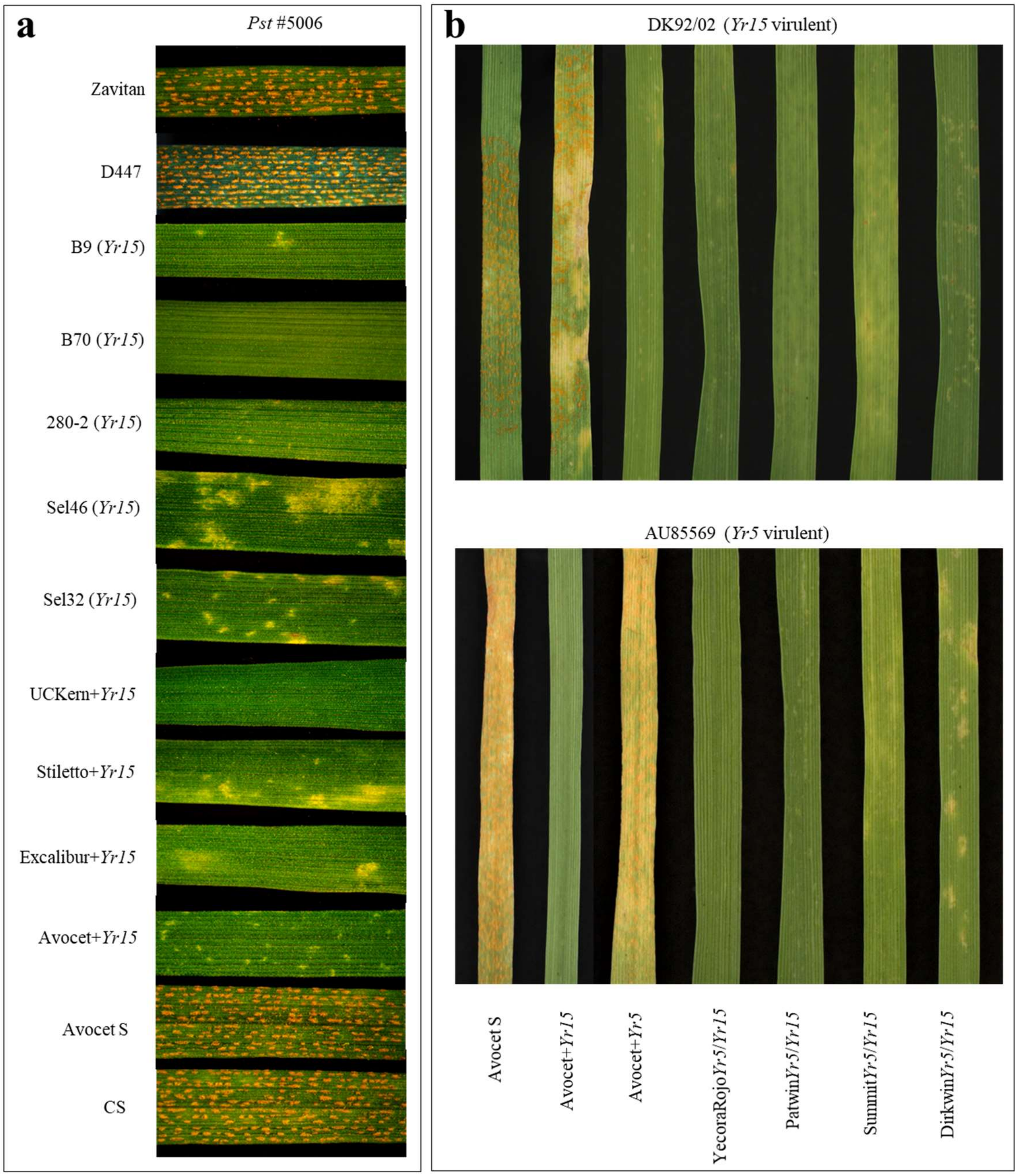

Supplementary Figure 1. a, Phenotyping of *Yr15* introgression lines[1] at 15 dpi, inoculated with *Pst* isolate #5006. All acceptor lines were previously shown to be susceptible to *Pst* isolate #5006[1]. Zavitan, D447, Avocet S and CS served as susceptible controls. **b**, Phenotypic responses of *Yr15, Yr5* and *Yr15/Yr5* introgression lines to inoculation with *Pst* isolates DK92/02 and AU85569 at 19 dpi. *Yr5* and *Yr15* were introgressed via marker-assisted selection (six backcrosses) respectively from donor lines Avocet+*Yr5* (Yr5/6*Avocet) and Sunset+*Yr15* (Yr15/6*Sunset) into the following recurrent parents (pedigrees in parentheses): YecoraRojo (Ciano 67//Sonora 64/Klein Rendidor/3/II-8156 = II23584), Patwin (Madsen/2*Express), Summit (Express//Tadorna/PB775), Dirkwin (Twin/Triple Dirk).

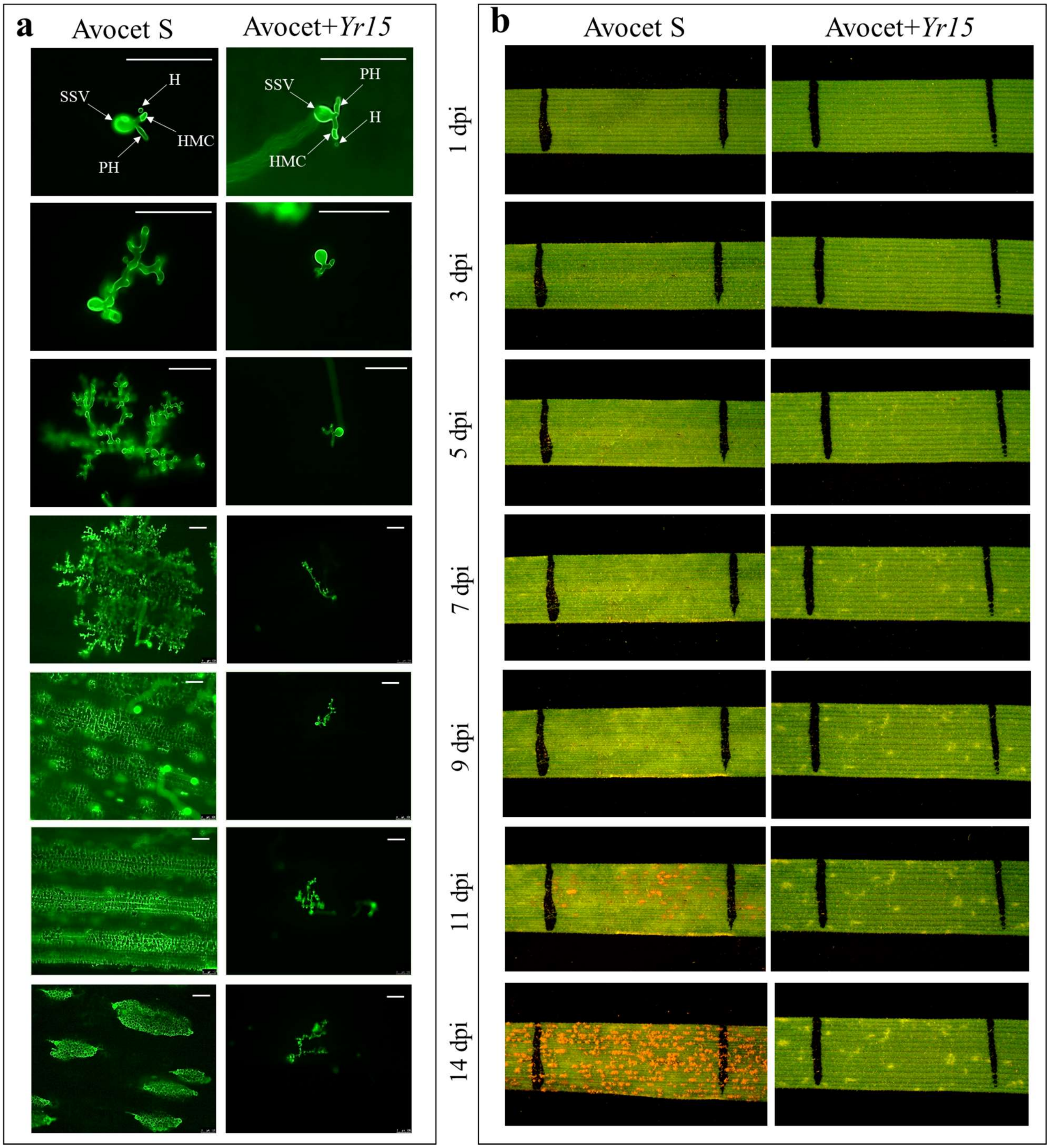

Supplementary Figure 2. Development of *Pst* within the leaves of susceptible Avocet S and resistant Avocet+*Yr15* NILs at different dpi. a, Fungal growth within leaf tissues of NIL host plants over 14 dpi. Scale bar is 100 µm; *SSV,* substomatal vesicle; *PH,* primary infection hyphae; *HMC,* haustorial mother cell; *H*, haustorium. **b**, Macroscopic observations of symptom development over 14 dpi, inoculated with *Pst* isolate #5006 in the susceptible line Avocet S as compared to the resistance response of its NIL Avocet+*Yr15*. The black lines delineate a 1 cm leaf segment in the middle of the second leaf of the same plant followed over 14 days.

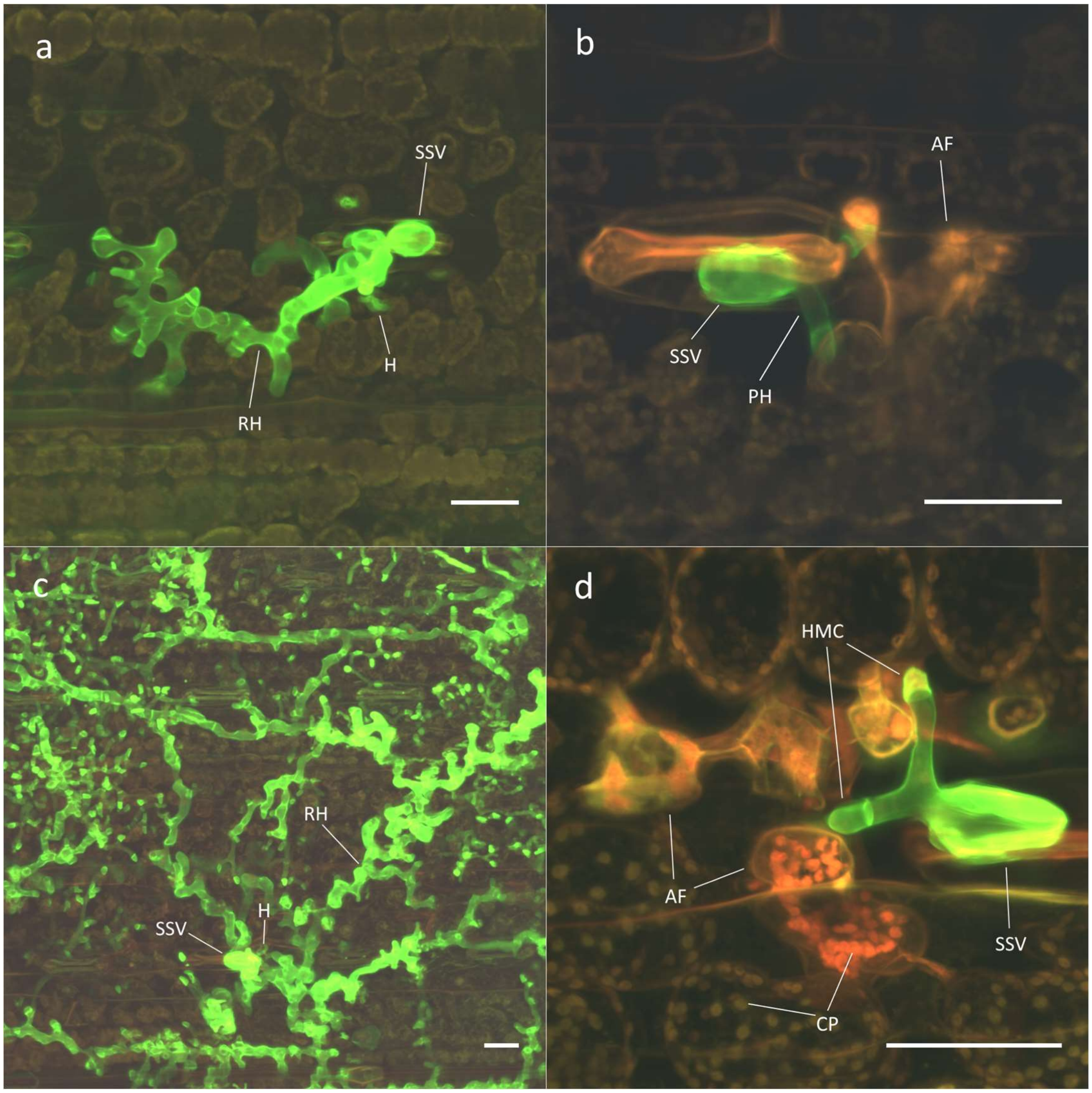

Supplementary Figure 3. Fungal structures and host responses in the susceptible Avocet S and resistant Avocet+*Yr15* NILs at 4 and 8 dpi with the *Pst* isolate UK75/30. Fungal structures were stained with the dye Uvitex2B and images acquired by confocal laser scanning microscopy. A 405 nm laser was used to visualize the fungal structures and host cell autofluorescence was recorded with a 515nm laser. **a**, A fungal colony (in bright green) in a leaf of Avocet S 4 dpi. A haustorium (H) is observed close to the substomatal vesicle (SSV) and the colony has started to develop runner hyphae (RH). **b**, A fungal colony in the resistant line Avocet+*Yr15* 4 dpi. The colony have developed two primary infection hyphae (PH), which are both associated with strong plant cell autofluorescence (AF) as a sign of a resistance response. **c**, Fungal structures in Avocet S 8 dpi. The colonies have developed extensive networks of RH containing several haustoria (H) without any signs of host cell autofluorescence. **d**, Host response and fungal structures in Avocet+*Yr15* 8 dpi. The fungal colony consists only of a SSV and two primary infection hyphae with HMCs. Some of the host cells show bright AF and appear to be collapsed and distorted, and a couple of them contain aggregated chloroplasts (CP) with bright orange fluorescence as an indicator of host HR. Bars = 40 µm.

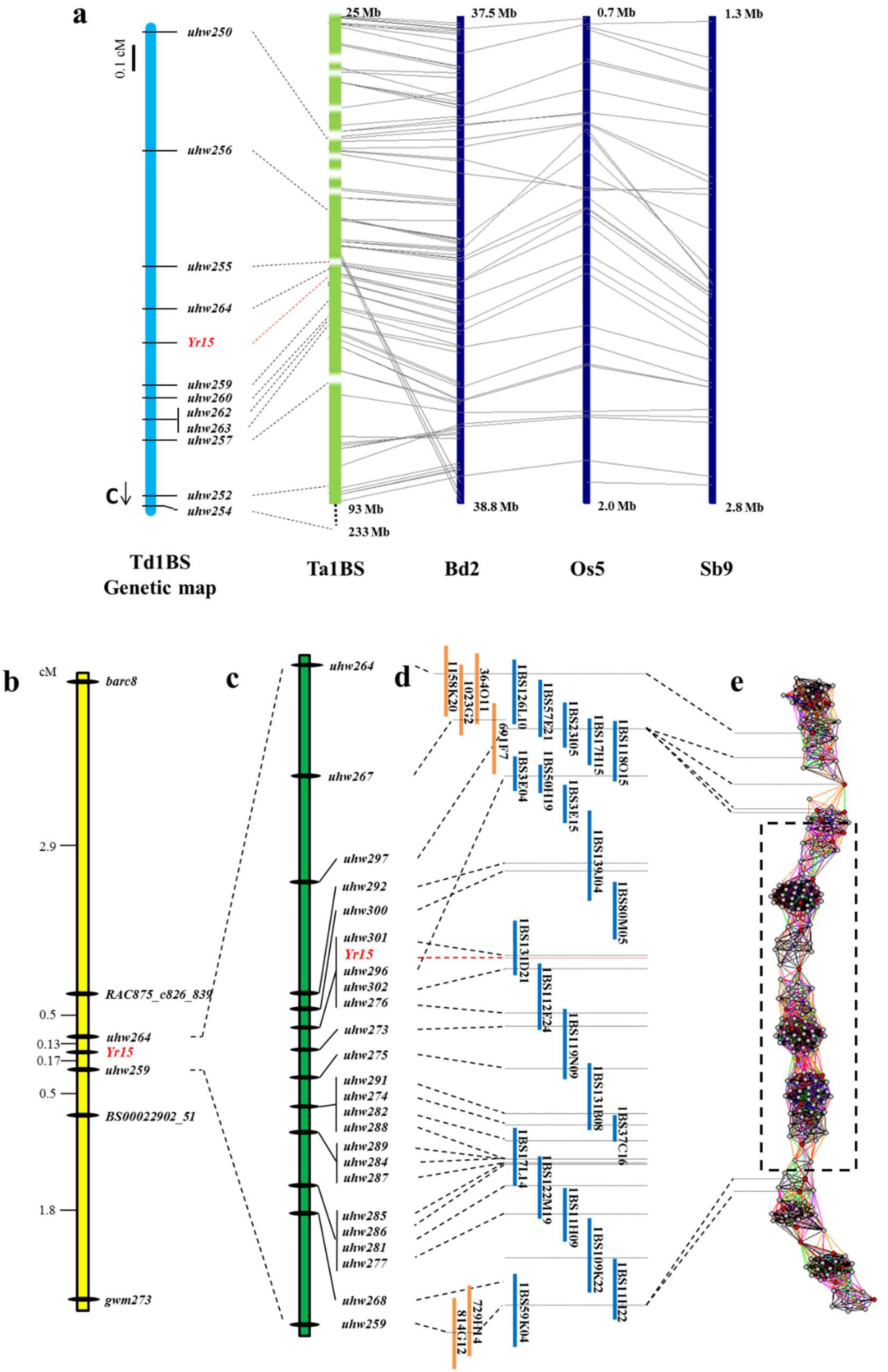

a
0.1 cM
uhw250
uhw256
uhw255
uhw264
Yr15
uhw259
uhw260
uhw262
uhw263
uhw257
C
uhw252
uhw254
25 Mb
93 Mb
233 Mb
37.5 Mb
38.8 Mb
0.7 Mb
2.0 Mb
1.3 Mb
2.8 Mb
Td1BS
Genetic map
Ta1BS
Bd2
Os5
Sb9
b
cM
barc8
2.9
RAC875_c826_839
0.5
uhw264
0.13
Yr15
0.17
uhw259
0.5
BS00022902_51
1.8
gwm273
c
uhw264
uhw267
uhw297
uhw292
uhw300
uhw301
Yr15
uhw296
uhw302
uhw276
uhw273
uhw275
uhw291
uhw274
uhw282
uhw288
uhw289
uhw284
uhw287
uhw285
uhw286
uhw281
uhw277
uhw268
uhw259
d
1158K20
1023G2
364O11
691F7
1BS126L10
1BS57F21
1BS23I05
1BS171I15
1BS118O15
1BS3E04
1BS50I19
1BS3E15
1BS139J04
1BS80M05
1BS131D21
1BS112E24
1BS119N09
1BS131B08
1BS37C16
1BS17L14
1BS122M19
1BS11H09
1BS109K22
1BS111I22
1BS59K04
729H4
814G12
e

Supplementary Figure 4. Genetic and physical maps of the *Yr15* region on chromosome arm 1BS. a, Collinearity of the wheat genetic and physical maps (Td1BS and Ta1BS, respectively) with *B. distachyon* (Bd2), *O. sativa* (Os5), and *S. bicolor* (Sb9). The genetic map of 1BS (blue bar) is based on the D447×B9 mapping population (scale in cM). The anchored CS 1BS (*T. aestivum*) scaffolds[2] are shown as green bars. **b**, The genetic map of the *Yr15* region, which is based on a mapping population of 302 F_2 plants from the D447×B9 cross. **c**, A high-resolution genetic map of the *Yr15* region, which is based on 8,573 F_2 plants, shows a 0.3 cM interval and the relative positions of markers close to *Yr15*. **d**, The order of the six CS BAC clones (orange) and 21 CS 1BS BAC clones (blue) that span the region corresponding to *Yr15* in DIC, as anchored by *uhw264* and *uhw259*[2]. The full names of the BAC clones contain the prefix "TaaCsp" (e.g., TaaCsp1BS126L10 for 1BS126L10). **e**, A part of the CS 1BS physical scaffold, ctg49, which harbors the *Yr15* region, and is presented as a network of clones (nodes) and clone overlaps (edges) that was assembled by the LTC software[2]; clones marked in red represent the minimal tiling path. Network structure shows that most of BACs here were proven by parallel clone overlaps that enabled to include these clones into contigs composing the scaffold. A single scaffold covering *Yr15* region enabled developing of markers used for positional cloning of *Yr15*.

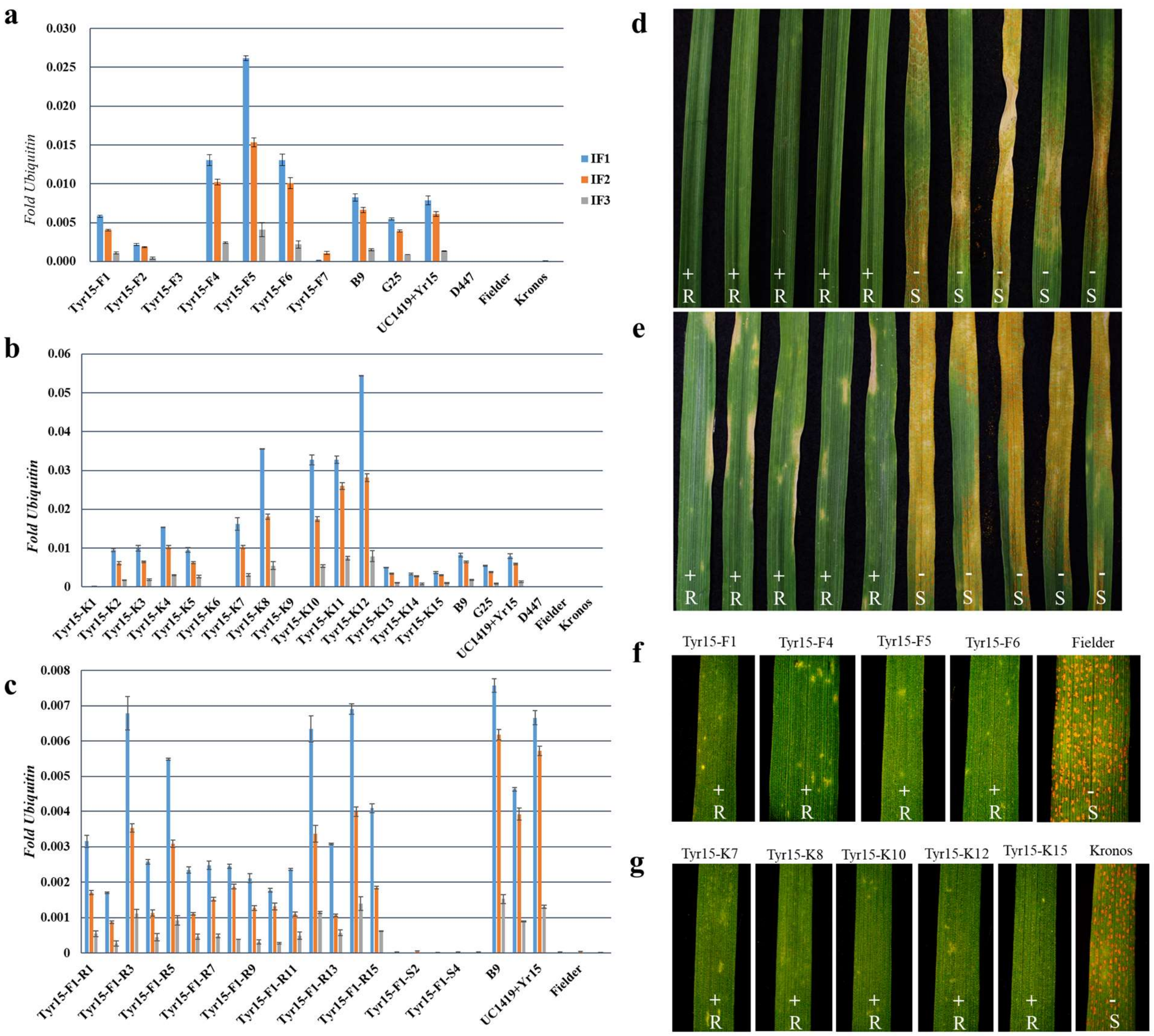

Supplementary Figure 5. Gain of function complementation. a-b, Transcript levels of the three isoforms (IF1, IF2, and IF3) of *Wtk1* in T_0 transgenic plants in the Fielder (a) background (Tyr15-F1～F7) and Kronos (b) background (Tyr15-K1～K15). Values are in fold-*Ubiquitin* levels calculated with the delta CT method. **c**, Transcript levels of the three isoforms of *Wtk1* in T_1 plants from transgenic event Tyr15-F1. Values are in fold-*Ubiquitin* levels. Error bars denoting s.e.m. are based on three technical replicates (a-c). **d-e**, Co-segregation of the *Wtk1* transgene with the resistance response on T_1 seedlings inoculated with *Pst* race PST-130 from events Tyr15-K8 (d) and Tyr15-F1 (e). Plants from Tyr15-K8 and Tyr15-F1 carrying *Wtk1* (+) showed a typical *Yr15*-mediated yellow rust resistance (R), while progenies without *Wtk1* (-), carrying only *wtk1*, were susceptible (S). **f-g**, Resistance phenotypes in T_2 *Wtk1* transgenic lines on Fielder (f) and Kronos (g) backgrounds inoculated with *Pst* isolate #5006.

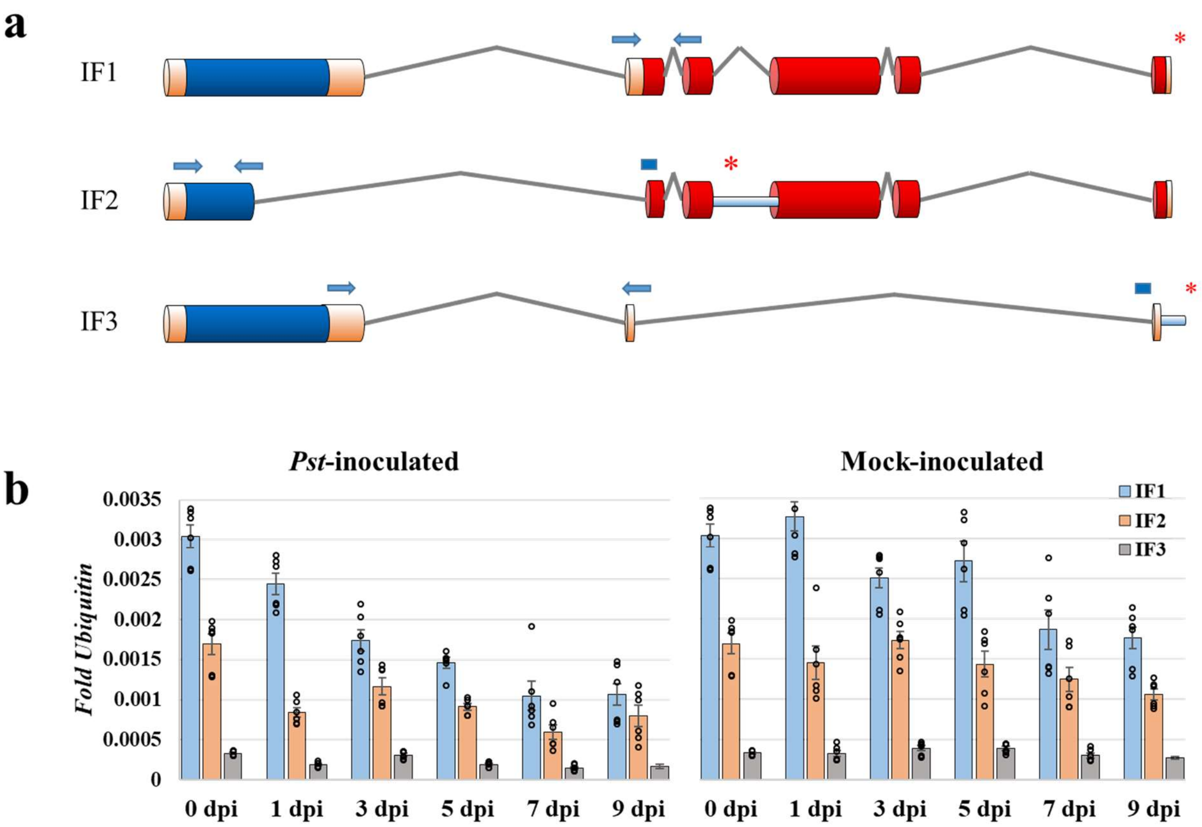

Supplementary Figure 6. *Wtk1* alternative splice forms. a, Isoform 1 (IF1), which encodes the complete WTK1 protein, is the main isoform, representing 85.7% of the sequenced *Wtk1* transcripts. Isoform 2 (IF2; 9.5%) results from a premature 5' donor splicing site in exon 1 (532 bp before the conserved GT splice site) and an alternative 3' splice acceptor site in exon 2 as well as the retention of intron 3. These differences in splicing generate a premature stop codon in intron 3. Isoform 3 (IF3; 4.8%) derives from the use of both an alternative 3' acceptor site in exon 2 (165 bp before the conserved GT splice site) and a 3' alternative acceptor site in exon 6 (80 bp after the conserved AG splice site), with a putative stop codon in the 3' UTR (77 bp after the conserved TGA). Color codes are as follows: KinI, blue; KinII, red; non-conserved protein parts, light orange. A red star indicates a stop codon. The arrows indicate the location of the primers that were used for expression analyses of the various splice forms. **b**, Transcript levels of the three isoforms of *Wtk1* in mock- or *Pst*-inoculated B9 plants. Error bars denote s.e.m. based on six biological and three technical replicates. The *p*-values for the differences between transcript levels of the three *Wtk1* isoforms in leaves of B9 inoculated with *Pst*, relative to mock-inoculated plants, tested by the homogeneity of slopes in GLM analysis, are: IF1, $p<0.00044$ (F-test, $df_1,df_2 = 1,60$), IF2, $p<0.00016$ (F-test, $df_1,df_2 = 1,60$), IF3, $p<0.0019$ (F-test, $df_1,df_2 = 1,60$). The *p*-values for the effect of time on expression over 1-9 dpi was calculated for each isoform separately: IF1, *Pst* inoculation ($p<8.0\times10^{-8}$, F-test, $df_1,df_2 = 1,30$) and mock inoculation ($p<2.4\times10^{-5}$, F-test, $df_1,df_2 = 1,30$); IF2, *Pst* inoculation ($p<0.22$, F-test, $df_1,df_2 = 1,30$) and mock inoculation ($p<0.023$, F-test, $df_1,df_2 = 1,30$); IF3, *Pst* inoculation ($p<0.033$, F-test, $df_1,df_2 = 1,30$) and mock inoculation ($p<0.11$, F-test, $df_1,df_2 = 1,30$).

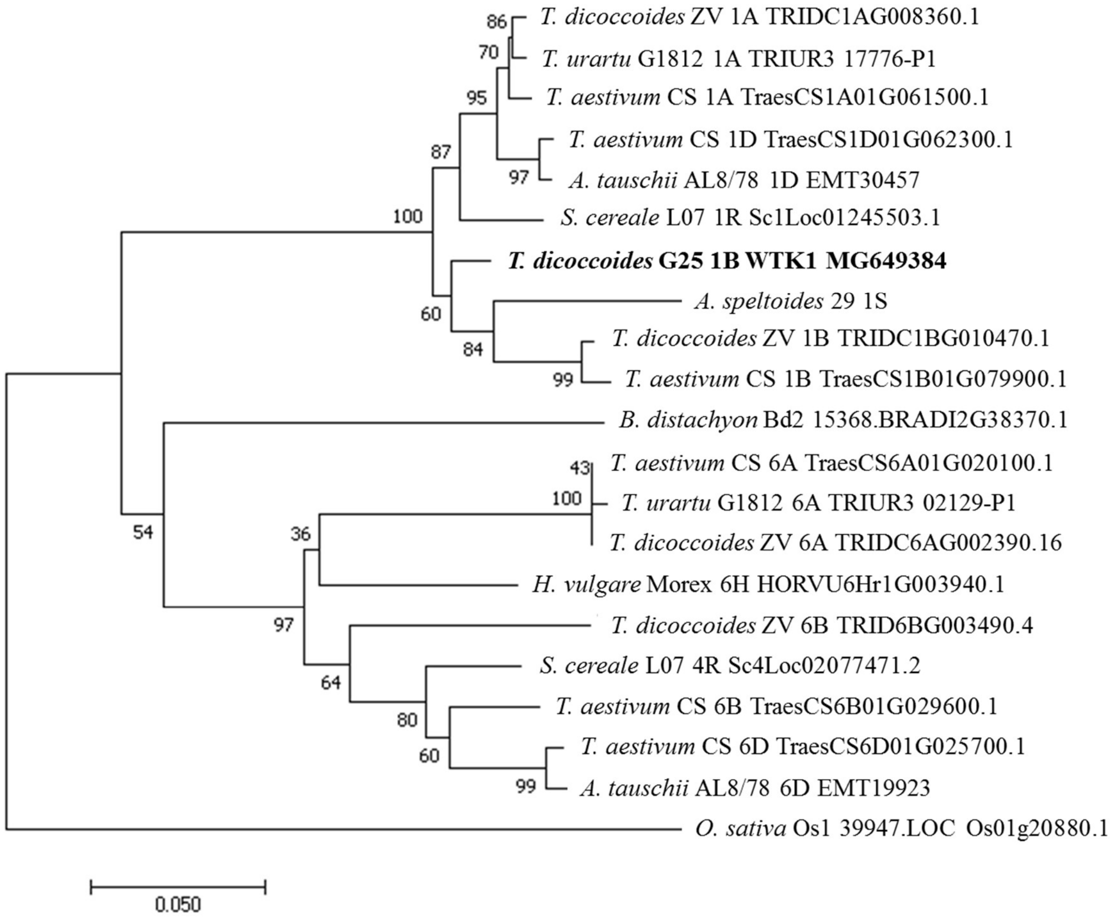

Supplementary Figure 7. Phylogeny of *WTK1* orthologs and paralogs in cereal species. The phylogeny of WTK1 was analyzed using the Neighbor-Joining algorithm, based on evolutionary distances calculated with the Poisson correction method[3]. Branches are labeled with the relevant plant species, abbreviations for the accession name, chromosome location and protein accession number. The numbers at nodes represent the percentage of replicated trees in which the associated taxa were clustered together in the bootstrap test (10,000 replicates).

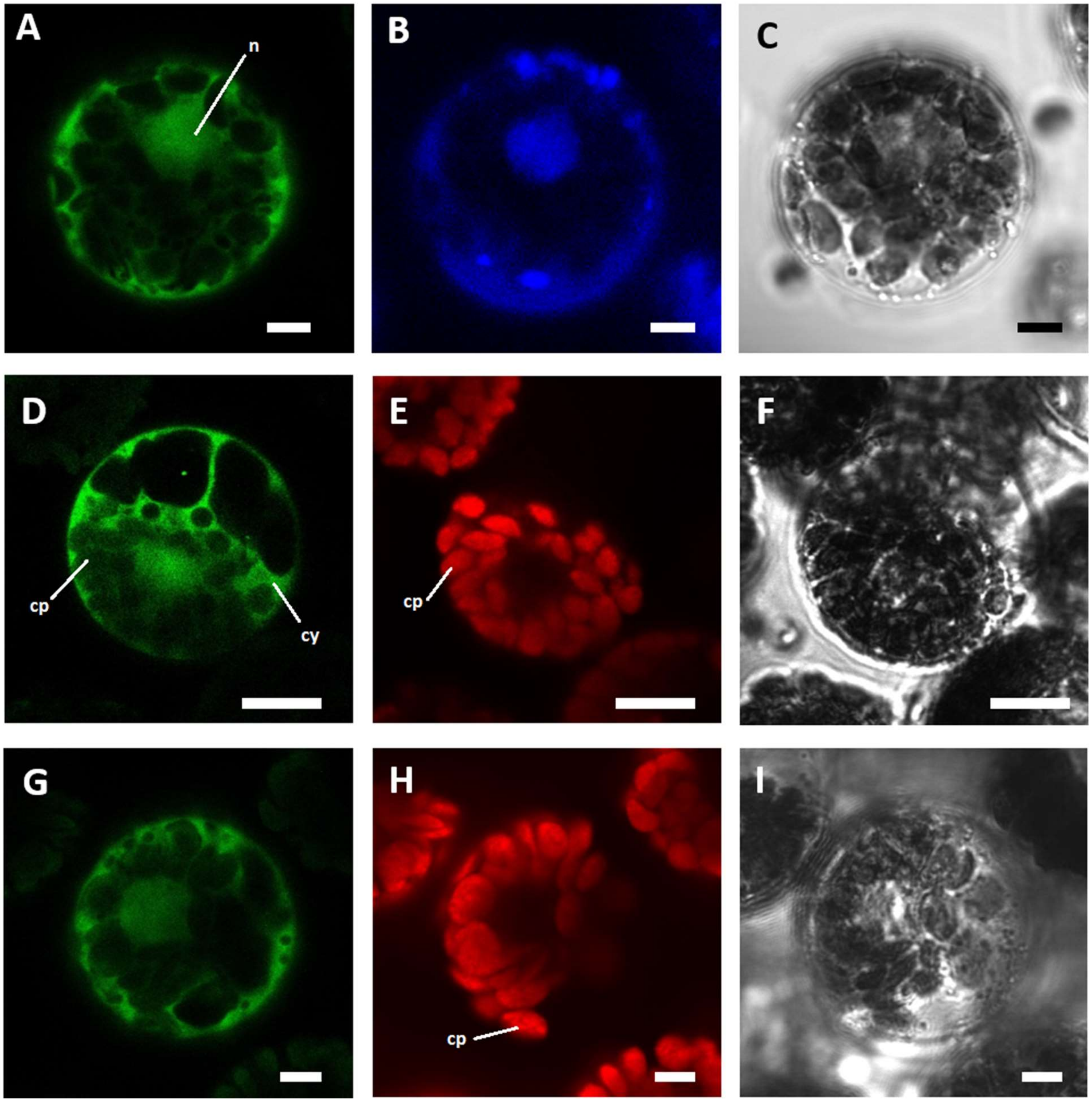

Supplementary Figure 8. Subcellular localization of WTK1 by transient transformation and expression in barley protoplasts. Fusions of GFP to the C-termini of (**A**) KinI, amino acids 1-326, (**D**) the KinII, amino acids 346-665, and (**G**) the entire WTK1 open reading frame expressed in barley protoplasts. (**B**) DAPI and (**E**, **H**) chloroplast auto-fluorescence of the cells in their respective rows. (**C**, **F**, **I**) bright-field visualization of the cells in their rows. Example chloroplasts (cp), cytoplasm (cy), and nuclei (n) are indicated. Scale bar is 5 μm except in (**D**), (**E**), and (**F**), where it is 10 μm.

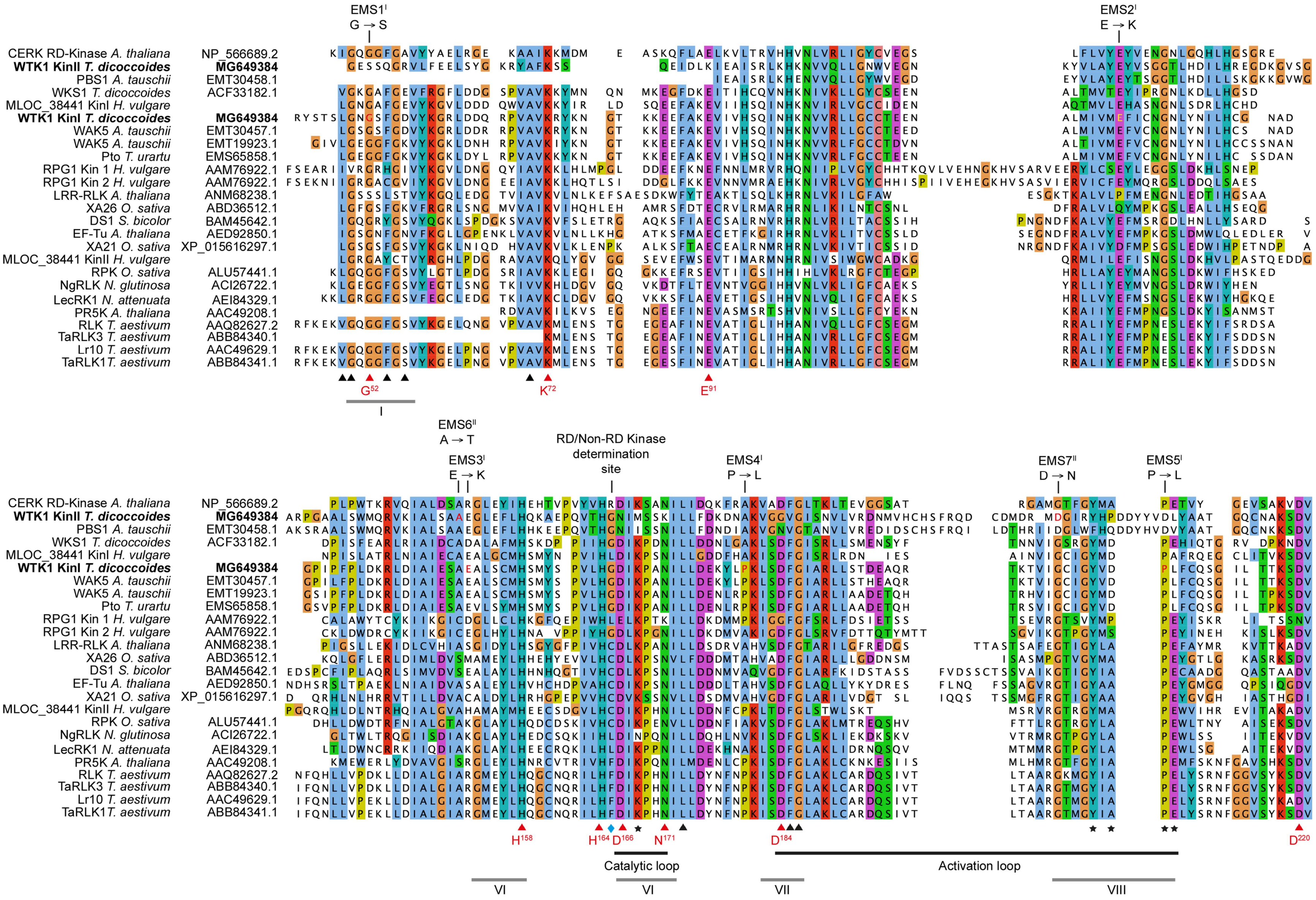
EMS1[I]
G → S
EMS2[I]
E → K
CERK RD-Kinase A. thaliana NP_566689.2
WTK1 KinII T. dicoccoides MG649384
PBS1 A. tauschii EMT30458.1
WKS1 T. dicoccoides ACF33182.1
MLOC_38441 KinI H. vulgare
WTK1 KinI T. dicoccoides MG649384
WAK5 A. tauschii EMT30457.1
WAK5 A. tauschii EMT19923.1
Pto T. urartu EMS65858.1
RPG1 Kin 1 H. vulgare AAM76922.1
RPG1 Kin 2 H. vulgare AAM76922.1
LRR-RLK A. thaliana ANM68238.1
XA26 O. sativa ABD36512.1
DS1 S. bicolor BAM45642.1
EF-Tu A. thaliana AED92850.1
XA21 O. sativa XP_015616297.1
MLOC_38441 KinII H. vulgare
RPK O. sativa ALU57441.1
NgRLK N. glutinosa ACI26722.1
LecRK1 N. attenuata AEI84329.1
PR5K A. thaliana AAC49208.1
RLK T. aestivum AAQ82627.2
TaRLK3 T. aestivum ABB84340.1
Lr10 T. aestivum AAC49629.1
TaRLK1 T. aestivum ABB84341.1
G52
K72
E91
I
EMS6[II]
A → T
EMS3[I]
E → K
RD/Non-RD Kinase
determination
site
EMS4[I]
P → L
EMS7[II]
D → N
EMS5[I]
P → L
H158
H164
D166
N171
D184
D220
Catalytic loop
Activation loop
VI
VI
VII
VIII

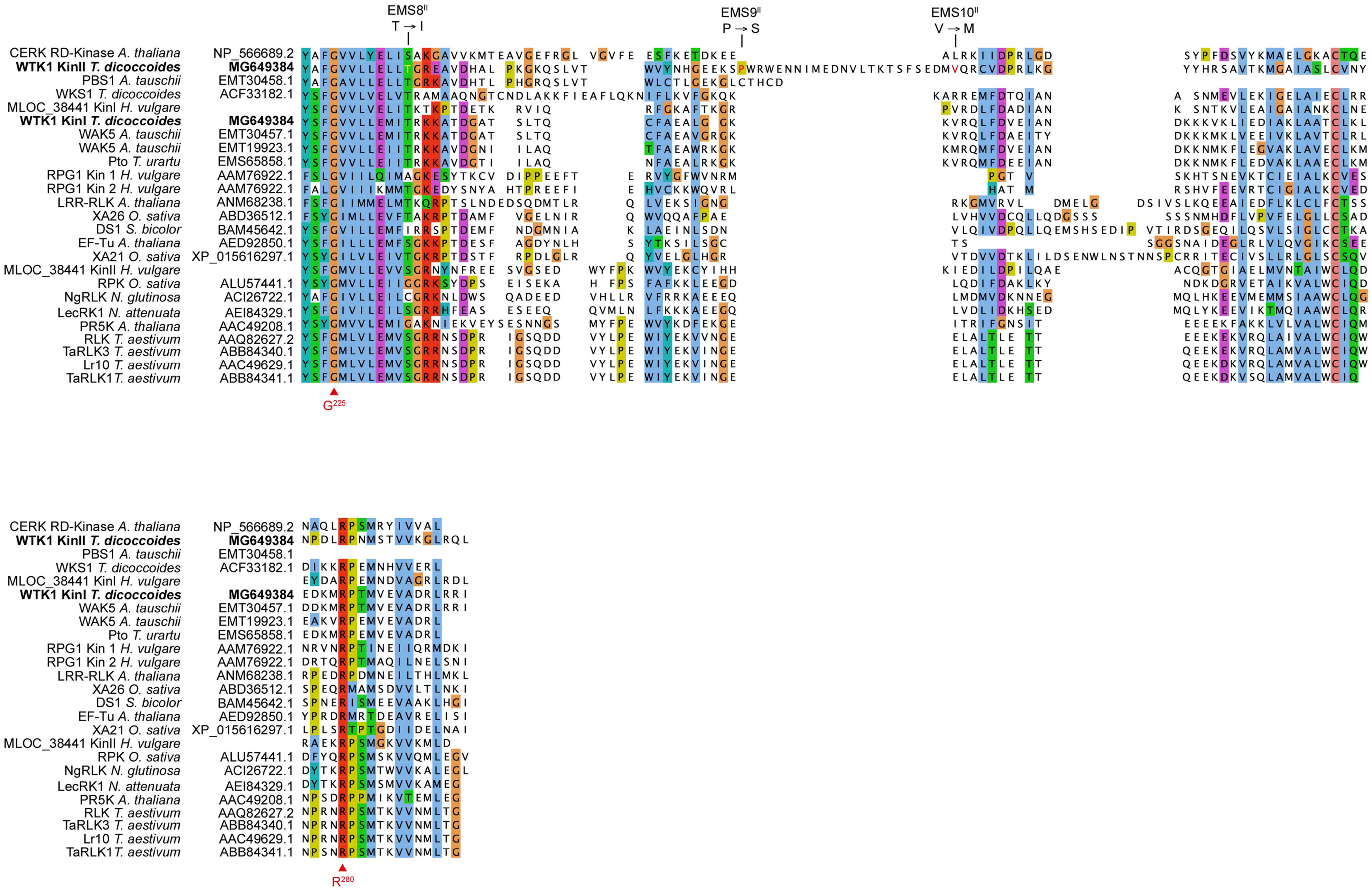
EMS8[II]
T → I
EMS9[II]
P → S
EMS10[II]
V → M
CERK RD-Kinase A. thaliana NP_566689.2
WTK1 KinII T. dicoccoides MG649384
PBS1 A. tauschii EMT30458.1
WKS1 T. dicoccoides ACF33182.1
MLOC_38441 KinI H. vulgare
WTK1 KinI T. dicoccoides MG649384
WAK5 A. tauschii EMT30457.1
WAK5 A. tauschii EMT19923.1
Pto T. urartu EMS65858.1
RPG1 Kin 1 H. vulgare AAM76922.1
RPG1 Kin 2 H. vulgare AAM76922.1
LRR-RLK A. thaliana ANM68238.1
XA26 O. sativa ABD36512.1
DS1 S. bicolor BAM45642.1
EF-Tu A. thaliana AED92850.1
XA21 O. sativa XP_015616297.1
MLOC_38441 KinII H. vulgare
RPK O. sativa ALU57441.1
NgRLK N. glutinosa ACI26722.1
LecRK1 N. attenuata AEI84329.1
PR5K A. thaliana AAC49208.1
RLK T. aestivum AAQ82627.2
TaRLK3 T. aestivum ABB84340.1
Lr10 T. aestivum AAC49629.1
TaRLK1 T. aestivum ABB84341.1
G225
R280

Supplementary Figure 9. Predicted WTK1 protein structure. Multiple alignment of 25 protein kinase domains used to infer the architecture of WTK1. In KinI (row 6), 13 out of the 14 key conserved residues of a protein kinase[4] are present. In the following description, these conserved residues are labelled with upper case numbers representing their position in the α form of the cAMP-dependent protein kinase catalytic subunit (cAPK). The nearly invariant residues of subdomain I (G-X-G^{52}-X-X-G) around the central G^{52} residue and subdomain VII (D^{184}, F^{185}, G^{186}) are conserved in WTK1 KinI, while the positions corresponding to cAPK A^{206} (D), P^{207} (P), and F^{208} (L) in subdomain VIII satisfy the consensus motif observed in 92 putative plant kinase domains (Fig. 4b). Based on the presence of a G residue in place of cAPK R^{165} in subdomain VI, WTK1 KinI was classified as a non-RD kinase. Furthermore, conserved residues in subdomains VI (cAPK D^{166} through N^{171}) and VIII (immediately upstream of cAPK A^{206}) indicate that WTK1 KinI is a serine/threonine protein kinase. In WTK1 KinII (row 2), 7 out of the 14 key conserved residues are strongly conserved and 5 positions have similar substitutions, placing it within the superfamily of protein kinase catalytic domains and therefore considered a putative pseudokinase. A fine-grained classification as RD or non-RD kinase or as serine/threonine or tyrosine kinase is not possible, because D^{166} has been substituted with an N residue and the diagnostic regions in subdomains VI and VIII are not conserved.
Labels: black triangles, ATP binding site; black stars, substrate binding site; red triangles, key conserved residues. The cyan diamond highlights the position within the catalytic loop that determines whether a kinase belongs to the RD (R, D; e.g. CERK in row 1) or non-RD (not R, D) group. Core conserved, diagnostic regions of subdomains I, VI, VII, and VIII are highlighted by grey bars labelled with Roman numerals. Amino acids altered by EMS mutagenesis that resulted in loss of WTK1 function are indicated by red or yellow letters. EMS mutagenized line designations, affected kinase domains (highlighted by ^{I} or ^{II}), and associated amino acid changes are indicated above the respective alignment column.

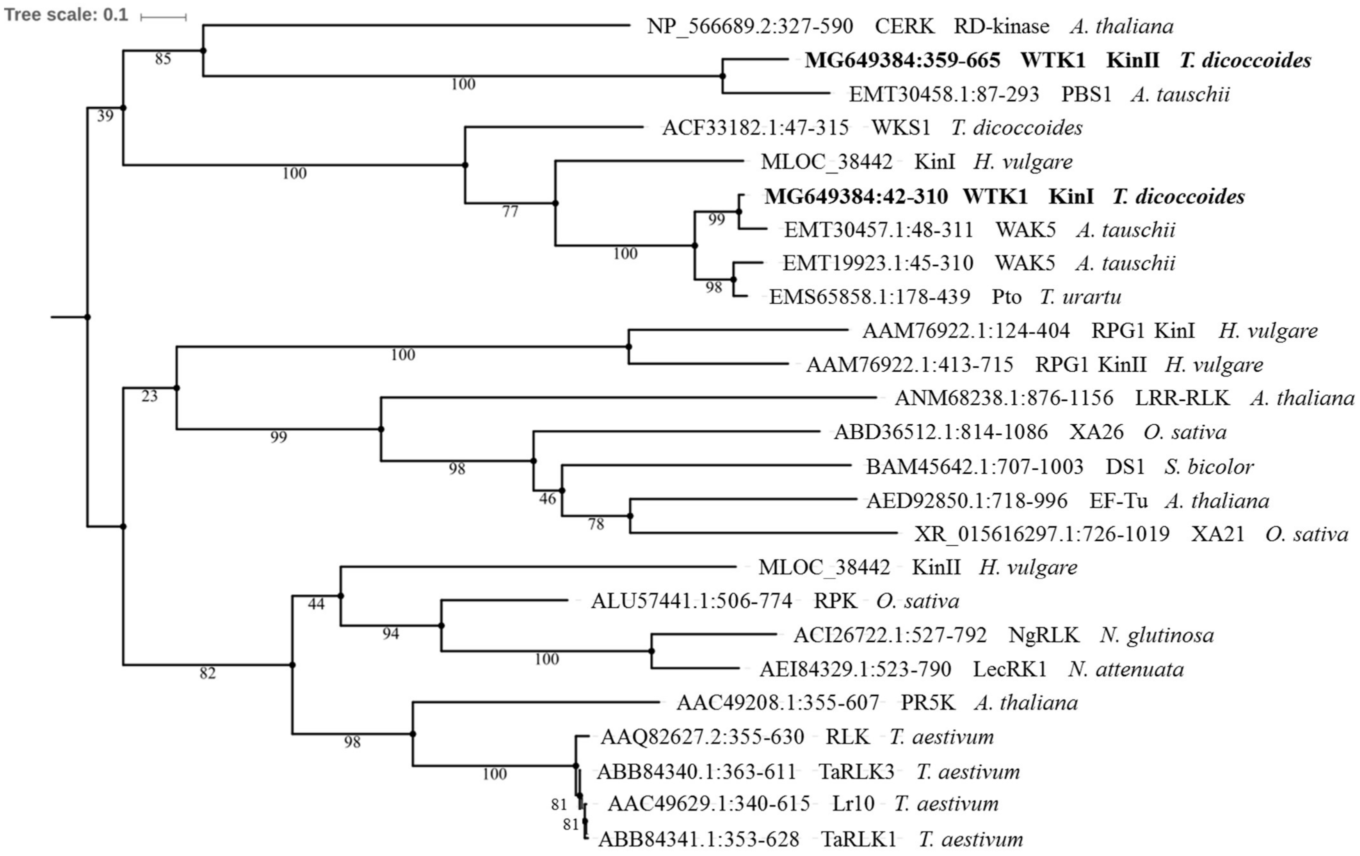

Supplementary Figure 10. Phylogenetic analysis of kinase domains from plant pattern recognition receptors (PRRs) and those predicted for WTK1. The phylogenetic tree is based on the kinase domain of each protein accession. The location of the domain is indicated by the sequence range numbers after the colon. The numbers at branch points represent the bootstrap values (% of 1,000 replicates). Branches are labeled with protein accession numbers, protein abbreviations, and the relevant plant species.

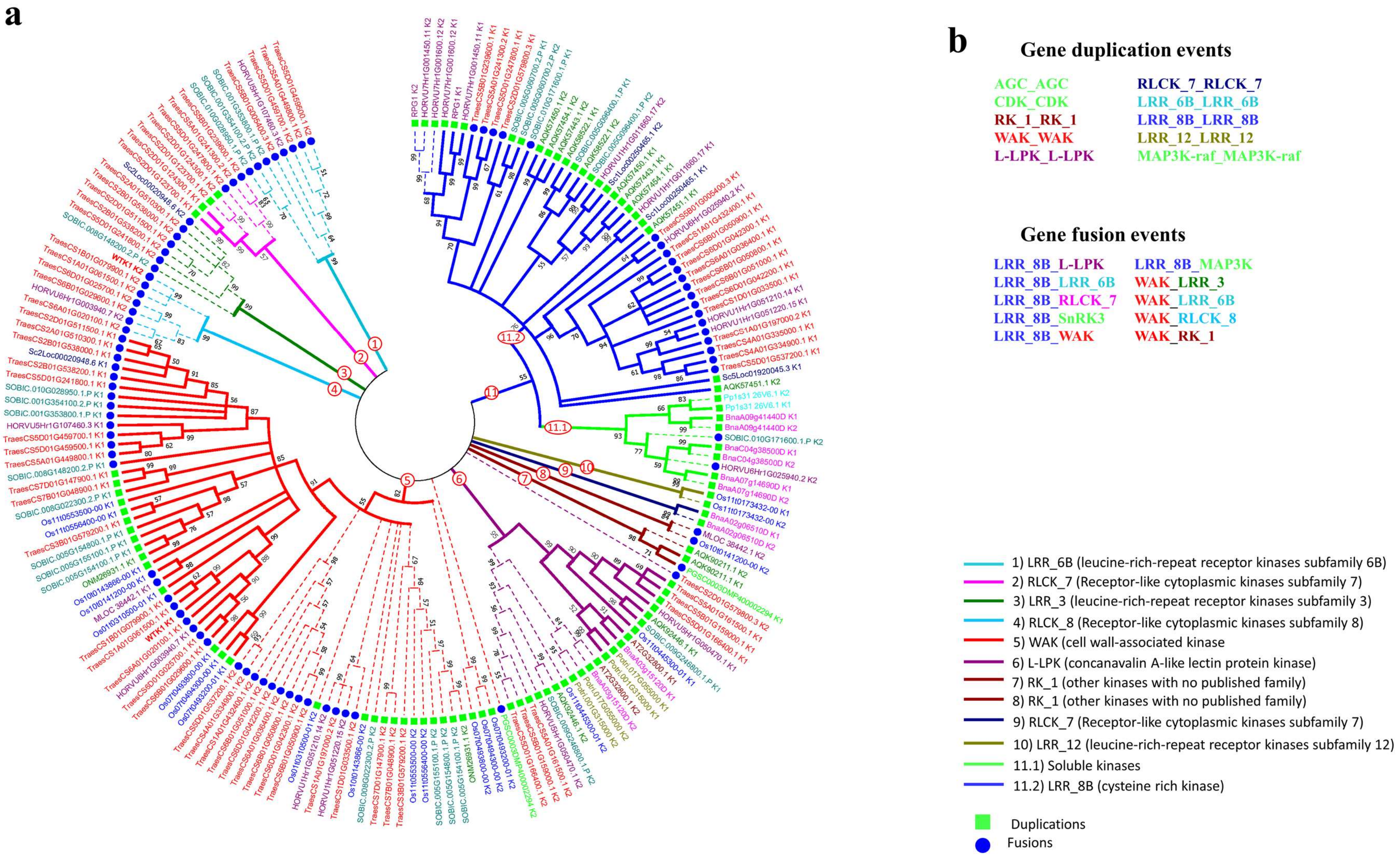
a
b
Gene duplication events
AGC_AGC
CDK_CDK
RK_1_RK_1
WAK_WAK
L-LPK_L-LPK
RLCK_7_RLCK_7
LRR_6B_LRR_6B
LRR_8B_LRR_8B
LRR_12_LRR_12
MAP3K-raf_MAP3K-raf
Gene fusion events
LRR_8B_L-LPK
LRR_8B_LRR_6B
LRR_8B_RLCK_7
LRR_8B_SnRK3
LRR_8B_WAK
LRR_8B_MAP3K
WAK_LRR_3
WAK_LRR_6B
WAK_RLCK_8
WAK_RK_1
1) LRR_6B (leucine-rich-repeat receptor kinases subfamily 6B)
2) RLCK_7 (Receptor-like cytoplasmic kinases subfamily 7)
3) LRR_3 (leucine-rich-repeat receptor kinases subfamily 3)
4) RLCK_8 (Receptor-like cytoplasmic kinases subfamily 8)
5) WAK (cell wall-associated kinase
6) L-LPK (concanavalin A-like lectin protein kinase)
7) RK_1 (other kinases with no published family)
8) RK_1 (other kinases with no published family)
9) RLCK_7 (Receptor-like cytoplasmic kinases subfamily 7)
10) LRR_12 (leucine-rich-repeat receptor kinases subfamily 12)
11.1) Soluble kinases
11.2) LRR_8B (cysteine rich kinase)
Duplications
Fusions

Supplementary Figure 11. Phylogenetic analysis of predicted tandem kinase-pseudokinase proteins from across the plant kingdom. a, Phylogram of 184 putative kinase domains of TKPs predicted from DNA sequences, which comprise 11 clades and 2 singletons. Each clade is similar to a specific family or sub-family of kinases from *Arabidopsis*[5]. KinI domains are labeled as K1 (solid line), and KinII domains as K2 (dash line).
Clade 1 is similar to LRR_6B (leucine-rich-repeat receptor kinase subfamily 6B). All of the domains in this clade are KinII domains and hence represent fusion TKPs.
Clade 2 is similar to RLCK_7 (receptor-like cytoplasmic kinase subfamily 7). All of the domains in this clade are members of wheat TKPs. This clade is composed of two well-supported nodes: one represents two TKPs derived from duplication events, and the second represents three KinII domains from fusion events.
Clade 3 is similar to LRR_3 (leucine-rich-repeat receptor kinase subfamily 3) and includes highly similar KinII domains from wheat, rye and sorghum.
Clade 4 is similar to RLCK_8 (receptor-like cytoplasmic kinase subfamily 8) and includes only KinII domains. One node comprised of 1BS WTK1 from WEW and orthologs of *T. aestivum* group 1 chromosomes, and a second node with paralogs of group 6 chromosomes of *T. aestivum* and *H. vulgare*.
Clade 5 is similar to the WAK family (cell wall-associated kinases) and includes a well-supported node with a cluster of KinI domains, four strongly supported nodes of KinII domains, and three singletons that are not resolved from the base of Clade 10.
Clade 6 is similar to L-LPK (concanavalin A-like lectin protein kinases). The clade derives from a single ancient duplication event (includes monocots and dicots) and contains two large sub-clades of KinI and KinII, respectively.
Clade 7 is related to RK_1 sub-family (other kinases with no published family) and includes one TKP from maize; and KinI of potato.
Clade 8 is similar to RK_1 sub-family (other kinases with no published family) and includes KinII of barley MLOC 36442 as well as its homolog from rice.
Clade 9 is similar to the RLCK_7 sub-family (receptor-like cytoplasmic kinase subfamily 7) and is composed of a single pair of domains from rapeseed.
Clade 10 is similar to the LRR_12 sub-family (leucine-rich-repeat receptor kinase subfamily 12) and contains one TKP comprised of very similar KinI-KinII pair from rice.
Clade 11 is comprised of two large clusters:
11.1 is similar to soluble kinases and includes members of five families: the MAP3K-raf sub-family (raf-like MAPK kinase kinase) from rapeseed and moss; the CDK family (cyclin dependent kinases); the AGC family (cATP-, cGTP- and phospholipid-dependent kinases) from rapeseed; as well as a KinII of the SnRK3 family (SNF1-related kinase 3) from barley and KinII of the MAP3K family (MAP3K kinase) from sorghum.
11.2 constitutes a large group of TKPs, corresponding to the LRR_8B sub-family (cysteine rich kinases). This cluster includes three singletons and five nodes. Singletons contain two KinI domains from rye and sorghum and KinII domain from maize. The largest node includes KinI domains from wheat, barley, rye, and maize. The next three nodes include a mix of KinI and KinII of four TKPs from maize, and single TKPs from barley, rye and sorghum. The last node includes the barley RPG1 KinI and KinII, their paralogs on chromosome 7H, and some homologs in wheat represented by KinI domains and both domains from sorghum.
The origins of sequences are coded as follows: Traes, wheat, *T. aestivum*; HORVU, barley, *H. vulgare*; Os, rice, *O. sativa*; AQK and ONM, maize, *Z. mays*; Sc, rye, *S. cereale*; SOBIC, sorghum, *S. bicolor*; PGSC, potato, *S. tuberosum*; Potri, black cottonwood, *Populus trichocarpa*; Bna, rapeseed or canola, *Brassica napus*; AT, *A. thaliana*; Pp, moss, *Ph. patens*.
b, Combinations of domains from kinase families and subfamilies resulted in tandem kinase-pseudokinase architecture of TKP family members.

Supplementary Table 1. Graphical genotyping and phenotyping of 13 selected RILs with the closest recombination events flanking *Yr15*. Homozygous RILs were tested for resistance to *Pst* isolate #5006. Marker alleles shown in yellow are identical to those obtained for the resistant parental line B9 (labeled B); marker alleles shown in green are identical to those obtained for the susceptible parental line D447 (D). Resistance (R) or susceptibility (S) to *Pst* is specified and highlighted, respectively, in yellow or green.

**Markers/ Parents and RILs**	***uhw264***	***uhw297***	***uhw292***	***uhw300***	***uhw301***	***Yr15***	***uhw296***	***uhw302***	***uhw276***	***uhw273***	***uhw275***	***uhw291***	***uhw274***	***uhw259***
B9	B	B	B	B	B	R	B	B	B	B	B	B	B	B
D447	D	D	D	D	D	S	D	D	D	D	D	D	D	D
B10-50-5-4	B	D	D	D	D	S	D	D	D	D	D	D	D	D
B10-52-54-8	D	B	B	B	B	R	B	B	B	B	B	B	B	B
B9-110-24-3	B	B	D	D	D	S	D	D	D	D	D	D	D	D
B9-14-14-7	D	D	B	B	B	R	B	B	B	B	B	B	B	B
B9-205-19-3	D	D	D	B	B	R	B	B	B	B	B	B	B	B
B9-L24-53-4	B	B	B	B	D	S	D	D	D	D	D	D	D	D
B9-167-105-1	D	D	D	D	D	S	D	D	D	B	B	B	B	B
B9-208-9-16	D	D	D	D	D	S	D	D	D	D	B	B	B	B
B9-183-45-1	B	B	B	B	B	R	B	B	B	B	B	D	D	D
B9-14-23-15	D	D	D	D	D	S	D	D	D	D	D	B	B	B
B9-L70-33-13	B	B	B	B	B	R	B	B	B	B	B	B	D	D
B9-167-63-1	B	B	B	B	B	R	B	B	B	B	B	B	B	D
B9-112-22-2	D	D	D	D	D	S	D	D	D	D	D	D	D	B

Supplementary Table 2. Description of PCR markers in the *Yr15* region developed from wheat ESTs assigned to deletion bin Sat0.31.

Marker name*	EST†	Primer sequence	Fragment size (bp)	Annealing temp (°C)	Restriction enzyme	Parental alleles (bp)
uhw250	BG275046	CTGCTCACTTTTTGCCTGTG	1355	50	-	B9: 1355
		AAAAGTTGTTGCTCTGCTTTT				D447: Null
uhw252	BG607503	GGTTTTCACTGAATCAATAGGG	550	57	*SspI*	B9: 552
		ATGAACCCAGCCAGTCTGAT				D447: 328, 222
uhw254	BG608205	CAATCCCAAGCTGACTGAAA	737	60	*AluI*	B9: 479, 258
		ACACCGCTGGTATTGTAGCC				D447: 737

*Marker order, from distal to proximal in relation to *Yr15*, is described in Supplementary Fig. 3a.
†ESTs assigned to deletion bin Sat0.31[6].

Supplementary Table 3. Description of markers in the *Yr15* region developed using collinearity to *B. distachyon*, *O. sativa*, and *S. bicolor*

Marker Name	EST / Unigene	Primer Sequence	Frag. Size (bp)	Annealing Temp. (°C)	Restr. Enzyme	Parental alleles (bp)	*B. distachyon* homolog	*O. sativa* homolog	*S. bicolor* homolog
uhw256	*HU35_455*	GTTACCCTCCACAGCAAGGT<br>GCGCATTACTTCCACTTCTTG	949	59	*AluI*	B9: 347, 310, 203, 60, 29<br>D447: 657, 203, 60, 29	*Bradi2g37690.1*	*LOC_Os05g02780.1*	*Sb09g001970*
uhw255	CJ661523	GATGCTCTGCACATGTGTTATG<br>GCAGCTCCAGCTTATTCGTC	753	55	*MseI*	B9: 243, 184, 150, 149, 71, 25<br>D447: 333, 243, 81, 71, 25	*Bradi2g37870.1*	*LOC_Os05g03100.1*	*Sb09g002120*
uhw264	CA730189	GGTCTCTTGCAACATACAGTAACAA<br>GAGTGGTAGTCTAGTAGAGGTTGGTG	247, 215	58		B9: 215<br>D447: 247	*Bradi2g37920.1*	*LOC_Os05g03120.1*	*Sb09g002140*
uhw259	CD927978	CTGTATTCTAATGCAGATTAGCTGTT<br>CACGCATAATTTTGTCCACAC	302	57	*BsrI*	B9: 112, 100, 90<br>D447: 190, 112	*Bradi2g37950.1*	*LOC_Os05g03140.1*	*Sb09g002180*
uhw260	CK155408	GAGACGAACGAAACCGAGAT<br>GATAGTGTCAATTGGTGGAAGG	381	58	*EcoRV*	B9: 300, 81<br>D447: 381	*Bradi2g37970.1*	*LOC_Os05g03174.1*	*Sb09g002200*
uhw262	BQ161309	TTTGCTTTGGAGTCCGAGTT<br>TTTGCAAAAGTAATCCTGGACA	915	59	*NlaIV*	B9: 915<br>DW1: 674, 241	*Bradi2g38030.1*	*LOC_Os05g03430.1*	*Sb09g002225*
uhw263	CJ841447	CACCAGTAGATCCTCAACAATACA<br>CTTGAGAATGGAATGCAACGTAG	539	58	*DraIII*	B9: 539<br>D447: 344, 195	*Bradi2g38030.1*	*LOC_Os05g03430.1*	*Sb09g002225*
uhw257	CJ582219	TCTTACTTGTTCACAAAACTGAGG<br>GCGCATGAGGAGCTTGAC	834	57		B9: Null<br>D447: 834	*Bradi2g38170.1*	*LOC_Os05g03610.1*	*Sb09g002320*

Supplementary Table 4. Description of KASP markers in the *Yr15* region developed based on SNPs from the wheat 15K SNP array.

Marker name	Primer sequence A	Primer sequence B	Primer sequence Common
RAC875_c826_839	ACGAAGGTTCTGTTTTCACCA	ACGAAGGTTCTGTTTTCACCG	TCTTCTTGCTCAAAGGTAAGAGT
BS00022902_51	ATGTGCGGCAGGAGAAGA	ATGTGCGGCAGGAGAAGG	ATACTCTTCACGGTCGTCTTC

Supplementary Table 5. Molecular markers developed from the physical map of chromosome arm 1BS of CS[2] and from the G25 BAC clone G25-64.

Marker Name	Marker Type	Sequence based design	Primer Sequences	Length (bp)	Conditions*	Enzyme	Parental alleles (bp)	1BS BAC clone†
uhw267	Genetic, co-dominant, CAPS	BES Sanger, BlastN EST CJ883493	TGGTAATCAAGTTTCACATTGTTCA GGAAGGACACCTTTCGGTATT	696	65/55; 0'40"	*NheI*	B9: 696 D447: 542, 172	TaaCsp1BS057E21
uhw297	Genetic, co-dominant, CAPS	PacBio, Uniprot	CAGATGACCAACCAAAAGCA GTCATATTGGTGCCCAGTGA	487	65/55; 0'40"	*BauI*	B9: 487 D447: 450, 37	TaaCsp1BS118O15
uhw292	Genetic, B9 dominant	BES IWGSC BLAST, BlasttX, Protein EMT24064	GACTTTCTTCCCTCGGGACT CTCGCACGCCTATAAAAGGA	1,000	65/55; 1'00"	-	B9: 1000 D447: null	TaaCsp1BS139J04
uhw296	Genetic, co-dominant, CAPS	PacBio, BlastX, Proteins EMS53293 and EMT11639	CAACCGTGCCTCCAAACA CGGGTGTTGTCCGTTGAG	497	65/55; 0'50"	*NlaIV*	B9: 321, 176 D447: 299, 176, 22	TaaCsp1BS050H19
uhw276	Genetic, co-dominant, CAPS	BES, 454, BlastX, Protein EMT12025	TCTGTGATGCCTGTGATGGT AAAGTTTGGGATTTGGCAAT	755	55; 0'40"	*TaqI*	B9: 309, 163, 73, 72, 71, 47, 20 D447: 182, 163, 127, 74, 73, 69, 47	TaaCsp1BS112E24
uhw273	Genetic, co-dominant, CAPS	BES, BlastX, Protein EMT27971, RGA	GGTGACGGCGAGTGTACG GACGCAATTGTCCGCTGT	242	62/52, 0'25"	*EagI*	B9: 149, 93 D447: 242	TaaCsp1BS112E24
uhw275	Genetic, co-dominant, CAPS	454, BlastX, Protein EMS52762	CAATGCTCGTAGCTGTTCCA GAGCATTGTTGGGGGTTG	549	65/55; 0'30"	*TaiI*	B9: 344, 205 D447: 344, 147, 58	TaaCsp1BS119N09
uhw291	Genetic, co-dominant, CAPS	454, ISBP Finder TE junction	TGTTGCTATGCCATCACCAT CTATACCGCTGGTGGGAGAA	285	65/55; 0'30"	*MspI*	B9: 142, 94, 49 D447: 143, 142	TaaCsp1BS131B08
uhw274	Genetic, co-dominant, CAPS	454, BlastX, Protein EMS64309	AAGCTCCGCTGCAATGAC ACCTGACATCCTCGAACCAC	314	65/55; 0'30"	*HaeIII*	B9: 196,118 D447: 314	TaaCsp1BS037C16
uhw282	Genetic, co-dominant, CAPS	454, BlastX, Protein EMT33303	TAATGTTTGGACGAGGCACA TTTTTACTTGTTGCTTGTTACAATTT	815	65/55; 0'40"	*AciI*	B9: 223, 214, 142, 130, 93, 10 D447: 359, 223, 130, 93, 10	TaaCsp1BS017L14
uhw288	Genetic, co-dominant, CAPS	454, ISBP Finder TE junction	GAGAGGGTTGACCTACGTGC GTGTATTGGAGTTGTGGCCC	1,567	65/55; 1'30"	*MseI*	B9: 377, 376, 304, 196, 172, 112, 24, 6 D447: 549, 376, 248 196, 112, 57, 24, 6	G25-64
uhw289	Genetic, co-dominant, CAPS	454, ISBP Finder TE junction	ATTTGCCTCTTCGCAATGAC TTTGCAAGAAACTCGCAATG	2,420	65/55; 1'30"	*SacII*	B9: 1984, 436 D447: 1191, 786, 436	G25-64

uhw280	Physical	454, BlastX, Protein BAJ96492	GCCTCCTGTACTAGCCGAAA<br>GCTAGGGTTCGGGTCTCTCT	183	65/55; 0'30”	-	B9: 183<br>D447: Null	TaaCsp1BS122M19
uhw284	Genetic, D447 dominant	454, ISBP Finder TE junction	TACTCTGTGCCTCTGGTCCC<br>GACGGAGACCTGCAAACAGT	279	60; 0'30”	-	B9: Null<br>D447: 279	TaaCsp1BS017L14
uhw287	Genetic, B9 dominant	454, GeneIous annotation, pathogen induced gene	CCTTACACCCCACGAATGAT<br>CGGACAGAGAGGAGACAAGG	388	63.5; 0'30”	-	B9: 388<br>D: Null	G25-64
uhw285	Genetic, co-dominant, CAPS	454, GeneIous annotation, heat shock induced gene	GCTGGAGGAGGAGGGAGAG<br>GCTTCTCCAGCTGCTTCACT	632	65/55; 0'30”	*AciI*	B9: 228, 114, 114, 57, 46, 40, 33<br>D447: 195, 114, 114 57, 46, 40, 33, 33	G25-64
uhw286	Genetic, co-dominant, CAPS	454, ISBP Finder TE junction[7]	CGGCAGTGGACTTCGTTACT<br>TTGCTGTCCCAAATTCATCA	194	65/55; 0'30”	*TaaI*	B9: 162, 32<br>D447: 86, 76, 32	G25-64
uhw281	Genetic, co-dominant, CAPS	454, BlastX, Protein AAM45953	GGCAGCCCAATACAGTTGTT<br>TTTTGCCGAATCAGAACTCC	243	65/55; 0'30”	*XceI*	B9: 244<br>D447: 140, 102	G25-64
uhw279	Physical	454, BlastX, Protein EMS57035	TTCACTCACGACAACTACCG<br>GGCCACCTCATCGCTGTC	399	65/55; 0'30”	-	B9: 399<br>D447: Null	TaaCsp1BS079P23
uhw277	Genetic, co-dominant, CAPS	454, BlastX, Protein AAP74646	GGGCTGTAGTCGCCTGTGTA<br>GGATATGCTGACGAGCCTTC	307	65/55; 0'20”	*DraIII*	B9: 176, 131<br>D447: 307	TaaCsp1BS079P23
uhw268	Genetic B9 dominant	BES Sanger, BlastN ESTs CD875071, CJ939425	AGGGCATCCAGGAATTTCA<br>AGCTGTAGTGCGACCTGGAG	994	60; 0'50”	-	B9: 994<br>D447: Null	TaaCsp729H14

*PCR annealing temperature (range is provided for touchdown) and the elongation time.
†Overlaps of CS 1BS BAC clones are presented in Supplementary Fig. 3d.

Supplementary Table 6. Molecular markers developed from the sequences of G25 BAC clone G25-141 and the Zavitan 1BS pseudomolecule.

Marker Name	Marker Type	Primer F sequence	Primer R sequence	Length
uhw300	Genetic, B9 dominant	CCGTGTCAGCCACCTACAAT	GCACTCTACCACCGAACACA	863 bp
uhw301	Genetic, B9 dominant	GTAGTGGCTCGTTCGGTGAT	TTTCGCATCCCACCCTACTG	936 bp
uhw302	Genetic, B9 dominant	CATCCATTCCTCCGACAAGT	CACTGCAATGCAAAAATGCT	216 bp

Supplementary Table 7. Molecular characterization of the *WTK1* EMS mutants.

Mutant No.	Protein domain in WTK1	Line ID	Base substitution[*]	Effect on amino acid[†]	SIFT[§]	Ploidy	Susceptible reaction to *Pst* inoculation[‡]
EMS1	KinI	Suncea+*yr15*-L18	G 160 A	G 54 S	**0.00**	6X	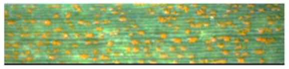
EMS2	KinI	Suncea+*yr15*-L89	G 340 A	E 114 K	**0.02**	6X	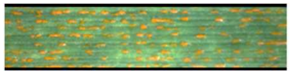
EMS3	KinI	B9+*yr15*-L1351	G 448 A	E 150 K	0.87	4X	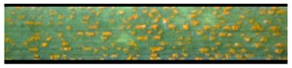
EMS4	KinI	Avocet+*yr15*-1	C 482 T	P 161 L	**0.00**	6X	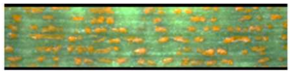
EMS5	KinI	Avocet+*yr15*-L90	C 632 T	P 211 L	**0.00**	6X	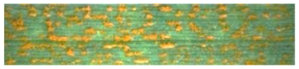
EMS6	KinII	Avocet+*yr15*-13	G 2922 A	A 460 T	**0.00**	6X	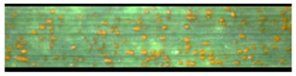
EMS7	KinII	Excalibur+*yr15*-6L306	G 3114 A	D 524 N	0.33	6X	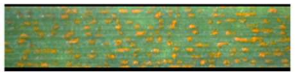
EMS8	KinII	Avocet+*yr15*-L72	C 3229 T	T 562 I	**0.00**	6X	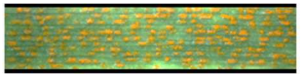
EMS9	KinII	B9+*yr15*-LF	C 3315 T	P 591 S	0.85	4X	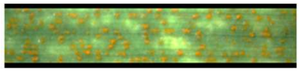
EMS10	KinII	Excalibur+*yr15*-L137	G 3469 A	V 614 M	0.14	6X	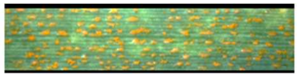

[*]The first letter indicates the wild-type nucleotide, the number indicates its position relative to the ATG start codon, and the last letter shows the mutant nucleotide. The complete *WTK1* coding regions of the above 10 mutants were sequenced; no additional mutations were detected.
[†]The first letter indicates the wild-type amino acid, the number indicates its position relative to the start methionine, and the last letter shows the mutant amino acid.
[‡]Yellow rust severity was evaluated at 14-18 dpi with *Pst* isolate #5006.
[§]The *WTK1* mutations were ranked using the SIFT program. Low SIFT scores (<0.05) predict mutations with severe effects on protein function.

Supplementary Table 8. Segregation ratio of resistance and susceptible phenotypes in F_2 families of EMS mutagenized lines carrying the *yr15* susceptible allele crossed with wild type Avocet+*Yr15* measured after inoculation with *Pst* isolate #5006.

Cross	F_2		Postulated ratio	χ^2 value	*P*-value*
	Resistant	Susceptible			
EMS4/Avocet+*Yr15*	82	24	3:1	0.31	0.29
EMS6/Avocet+*Yr15*	77	28	3:1	0.16	0.35

**P*-value for a one-tail test of significance by χ^2 statistics with *df*=1

Supplementary Table 9. PCR primers used for the functional characterization of *WTK1*.

	Function	Primer name	Primer sequence
**Transgenics**		Yr15F1	CACCCTCGAGCGAGGTGGTCGTCCAGTAGTT
		Yr15R1	CTAAGGAGACTACAGACCTAACAT
		Yr15F2	CACCCAGTAGGGTGGGATGCGAAATA
		Yr15R2	CTTTACCTAGGTTCTCTCTCCATCCCAACCAAT
		HpyF1	GGCCTCCAGAAGAAGATGTTGG
		HpyR1	GAGCCTGACCTATTGCATCTCC
		Yr15TestF1	TGGGTCGAAGGAAACAAATA
		Yr15TestR1	AACAGCACTGCGATGGTAATA
		Y15K1_F2	GGAGATAGAGCACATTACAGAC
		uhw301R	TTTCGCATCCCACCCTACTG
**Transcription**	qRT-PCR	Ubiquitin_F	TTGACAACGTGAAGGCGAAG
		Ubiquitin_R	GGCAAAGATGAGACGCTGCT
	qRT-PCR	Isoform1_F	GAAATTGATTTGAAGATTGAAGCA
		Isoform1_R	CATATTTGTTTCCTTCGACCCAA
	qRT-PCR	Isoform2_F	GTGGCAATGCAGATGGTCCT
		Isoform2_R	TTGAGAAGACTTGATGGCGATGTC
	qRT-PCR	Isoform3_F	TCCGACAATGGTTGAGGTAGC
		Isoform3_R	GCAACAATTGCCTTTCCTGTTC
	Full-length cDNA	WTK1_L2F	CTGCTGCTACCTGTTCTGTAA
		WTK1_RE6	ACAGACAGTGACACGGACAT
		E1820	AAGCAGTGGTAACAACGCAGAGTACTTTTTTTTTTTT TTTTTTTTTTTTTTTTTNA
		E2146	AAGCAGTGGTAACAACGCAGAGTAC
		Y15F0	CAGGCAGGCTGCTGCTAC
		Y15F2	GTCTTCATATGCTGCTTGCAC
		RNAoligo	GCUGAUGGCGAUGAAUGAACACUG
		5'RACE	GCTGATGGCGATGAATGAACACTG
		Y15R2	TGCTTCCGCTGACTCGATGGC
		Y15R1	TGCAGCAGCCTAACAATCTG
**Subcellular localization**		attB4F35S	GGGGACAACTTTGTATAGAAAAGTTGAGATTAGCCTT TTCAATTTCAG
		attB1R35S	GGGGACTGCTTTTTTGTACAAACTTGCGTGTTCTCTCC AAATGAAATG
		attB1FKinase	GGGGACAAGTTTGTACAAAAAAGCAGGCTCCRCCATG GATTACCAAGG
		attB2Rkinase	GGGGACCACTTTGTACAAGAAAGCTGGGTGGTTGATG CCTGTAGAGC
		attB2FGFPnos	GGGGACAGCTTTCTTGTACAAAGTGGGCATGGTGAGC AAGGGCGAGGAGC
		attB3RGFPnos	GGGGACAACTTTGTATAATAAAGTTGCATCTAGAGGG CCCGGATCTG
		attB1Freg	GGGGACAAGTTTGTACAAAAAAGCAGGCTCCRCCATG AAGAAACTAACAAG
		attB2Ryr15	GGGGACCACTTTGTACAAGAAAGCTGGGTGCTTTTGC AACAATTGCCTC
**EMS mutant screen**		WJKDF1	GCTGCTGCTACCTGTTCTGT
		WJKDR1	CCCTACCTAATATCCGCGTGC
		WJKDF2	TCCCAATCCTTGTGTGCTACC
		WJKDR2	TTGCCCAGGATTCACCACTA
		WJKDF3	GCTGCTGGAGCTTTTAACCG
		WJKDR3	GCGAGAGAACGATTTCCCCT
**Germplasm screen**	KinI	Y15K1_F2	GGAGATAGAGCACATTACAGAC
		uhw301R	TTTCGCATCCCACCCTACTG
	KinII	W_2F	TGCACGCGGATATTAGGTAGG
		W_2R	TGATGAAGAGGACCAACGCA

Supplementary Table 10. DIC accessions evaluated for the presence of *Wtk1*. Each accession was evaluated with a set of two diagnostic markers designed to amplify the two conserved domains of *Wtk1* (Supplementary Table 9).

Wheat	No.	*Wtk1*	Accession number*
DIC southern population†	29	+	TD103986, TD104084, TD104266, TD104326, TD104422, TD104424, TD104430, TD104432, TD104447, TD104668, TD104732, TD104733, TD104785, PI233288, PI428100, PI428126, PI428127, PI428132, PI428135, PI428143, PI466991, PI503314, PI538690, PI538700, PI503316, IG46526, IG46504, CGN19932, CGN21078
	133	-	TD103984, TD103989, TD104006, TD104015, TD104019, TD104020, TD104541, TD104542, TD104064, TD104068, TD104069, TD104073, TD104088, TD104089, TD104093, TD104134, TD104136, TD104139, TD104140, TD104144, TD104552, TD104555, TD104157, TD104159, TD104168, TD104172, TD104173, TD104177, TD104183, TD104191, TD104205, TD104213, TD104214, TD104559, TD104247, TD104249, TD104253, TD104255, TD104257, TD104261, TD104263, TD104268, TD104279, TD104290, TD104293, TD104294, TD104302, TD104306, TD104336, TD104375, TD104397, TD104404, TD104409, TD104413, TD104425, TD104429, TD104434, TD104446, TD104449, TD104453, TD104455, TD104463, TD104465, TD104466, TD104467, TD104469, TD104470, TD104531, TD104597, TD104599, TD104600, TD104619, TD104621, TD104628, TD104684, TD104727, TD104742, TD104751, TD104755, TD104757, TD104764, TD104766, TD104767, TD104768, TD104774, PI414719, PI414720, PI414721, PI414722, PI428014, PI428093, PI428097, PI428099, PI428105, PI428119, PI466949, PI466955, PI466981, PI467004, PI471016, PI471035, PI487253, PI487255, PI503312, PI503315, PI538680, PI538684, PI538685, PI538699, PI538719, IG46439, IG46457, IG46473, IG46466, IG46492, IG46476, IG45490, IG45493, IG45494, IG45500, IG45502, IG45676, IG45726, IG45964, IG46320, IG46323, IG46324, IG46352, IG46386, IG46397, IG46420, IG110737, IG110815
DIC northern population‡	19	-	PI428017, PI428018, PI428053, PI428054, PI428069, PI428077, PI428092, PI538626, PI538633, PI538651, PI538656, PI538657, PI554580, PI554582, PI554583, PI503310, PI428016, IG116173, IG116184

*DIC accessions were obtained from the National Small Grains collection (NSGC, USDA; PI numbers), the Wild Cereal Gene Bank (ICGB; Institute of Evolution, University of Haifa; TD numbers)[8], the International Center for Agricultural Research in Dry Areas (ICARDA; Rabat, Morocco; IG numbers), the Center for Genetic Resources (CGN; Wageningen University & Research; CGN numbers).

†Wild emmer wheat originating from Israel, Lebanon, Jordan, or Syria[9].

‡Wild emmer wheat originating from Turkey or Iran[9].

Supplementary Table 11. *Triticum* and *Aegilops* species evaluated for the presence of *Wtk1*. Each accession was screened by PCR for the KinI and KinII regions of *Wtk1* with gene-specific diagnostic markers (Supplementary Table 9).

Germplasm number/variety number	genus	species	subspecies	KinI	KinII
CGN4219	*Triticum*	*turgidum*	*dicoccum*	-	-
CGN6542	*Triticum*	*turgidum*	*dicoccum*	-	-
CGN6543	*Triticum*	*turgidum*	*dicoccum*	-	-
CGN7965	*Triticum*	*turgidum*	*dicoccum*	-	-
CGN7966	*Triticum*	*turgidum*	*dicoccum*	-	-
CGN7975	*Triticum*	*turgidum*	*dicoccum*	-	-
CGN8031	*Triticum*	*turgidum*	*dicoccum*	-	-
CGN8104	*Triticum*	*turgidum*	*dicoccum*	-	-
CGN8344	*Triticum*	*turgidum*	*dicoccum*	-	-
CGN8345	*Triticum*	*turgidum*	*dicoccum*	-	-
CGN8348	*Triticum*	*turgidum*	*dicoccum*	-	-
CGN8350	*Triticum*	*turgidum*	*dicoccum*	-	-
CGN8356	*Triticum*	*turgidum*	*dicoccum*	-	-
CGN8362	*Triticum*	*turgidum*	*dicoccum*	-	-
CGN10424	*Triticum*	*turgidum*	*dicoccum*	-	-
CGN10425	*Triticum*	*turgidum*	*dicoccum*	-	-
CGN11482	*Triticum*	*turgidum*	*dicoccum*	-	-
CGN11486	*Triticum*	*turgidum*	*dicoccum*	-	-
CGN12275	*Triticum*	*turgidum*	*dicoccum*	-	-
CGN12278	*Triticum*	*turgidum*	*dicoccum*	-	-
CGN16105	*Triticum*	*turgidum*	*dicoccum*	-	-
CGN21064	*Triticum*	*turgidum*	*dicoccum*	-	-
CGN21069	*Triticum*	*turgidum*	*dicoccum*	-	-
CGN12283	*Triticum*	*turgidum*	*dicoccum*	-	-
CGN13154	*Triticum*	*turgidum*	*dicoccum*	-	-
PI532302	*Triticum*	*turgidum*	*dicoccum*	-	-
PI319868	*Triticum*	*turgidum*	*dicoccum*	-	-
PI319869	*Triticum*	*turgidum*	*dicoccum*	-	-
PI355454	*Triticum*	*turgidum*	*dicoccum*	-	-
PI355496	*Triticum*	*turgidum*	*dicoccum*	-	-
PI352357	*Triticum*	*turgidum*	*dicoccum*	-	-
PI352348	*Triticum*	*turgidum*	*dicoccum*	-	-
PI352367	*Triticum*	*turgidum*	*dicoccum*	-	-
PI352352	*Triticum*	*turgidum*	*dicoccum*	-	-
PI182743	*Triticum*	*turgidum*	*dicoccum*	-	-
PI352361	*Triticum*	*turgidum*	*dicoccum*	-	-
PI191091	*Triticum*	*turgidum*	*dicoccum*	-	-
PI276007	*Triticum*	*turgidum*	*dicoccum*	-	-
PI606325	*Triticum*	*turgidum*	*dicoccum*	-	-
PI352329	*Triticum*	*turgidum*	*dicoccum*	-	-
PI94741	*Triticum*	*turgidum*	*dicoccum*	-	-
PI377658	*Triticum*	*turgidum*	*dicoccum*	-	-
PI264964	*Triticum*	*turgidum*	*dicoccum*	-	-
PI470739	*Triticum*	*turgidum*	*dicoccum*	-	-
PI94661	*Triticum*	*turgidum*	*dicoccum*	-	-
PI470737	*Triticum*	*turgidum*	*dicoccum*	-	-
PI326312	*Triticum*	*turgidum*	*dicoccum*	-	-
PI94640	*Triticum*	*turgidum*	*dicoccum*	-	-
CGN4222	*Triticum*	*turgidum*	*carthlicum*	-	-
CGN6596	*Triticum*	*turgidum*	*carthlicum*	-	-
CGN8389	*Triticum*	*turgidum*	*polonicum*	-	-
CGN8391	*Triticum*	*turgidum*	*polonicum*	-	-
CGN12289	*Triticum*	*turgidum*	*polonicum*	-	-
CGN12291	*Triticum*	*turgidum*	*polonicum*	-	-

CGN12293	*Triticum*	*turgidum*	*polonicum*	-	-
CGN4224	*Triticum*	*monococcum*		-	-
CGN6598	*Triticum*	*monococcum*		+	-
CGN6602	*Triticum*	*monococcum*		+	-
CGN9956	*Triticum*	*monococcum*		-	-
CGN10500	*Triticum*	*monococcum*		-	-
CGN10682	*Triticum*	*speltoides*		-	-
CGN10684	*Triticum*	*speltoides*		-	-
CGN10686	*Triticum*	*speltoides*		-	-
CGN10687	*Triticum*	*speltoides*		-	-
CGN10692	*Triticum*	*speltoides*		+	-
CGN10697	*Triticum*	*speltoides*		-	-
CGN10698	*Triticum*	*speltoides*		-	-
CGN10671	*Triticum*	*speltoides*	*speltoides*	-	-
CGN10673	*Triticum*	*bicorne*		-	-
CGN10672	*Triticum*	*bicorne*		-	-
CGN6525	*Triticum*	*triunciale*		+	-
CGN6604	*Triticum*	*triunciale*		+	-
CGN10660	*Triticum*	*triunciale*		+	-
CGN8408	*Triticum*	*timopheevii*	*timopheevii*	-	-
CGN10495	*Triticum*	*timopheevii*	*timopheevii*	-	-
CGN10497	*Triticum*	*timopheevii*	*timopheevii*	-	-
CGN10677	*Triticum*	*longissimum*	*longissima*	-	-
CGN10681	*Triticum*	*longissimum*	*longissima*	-	-
CGN10777	*Triticum*	*longissimum*	*sharonensis*	-	-
CGN16088	*Triticum*	*longissimum*	*sharonensis*	-	-
CGN13124	*Triticum*	*longissimum*	*sharonensis*	-	-
CGN13111	*Triticum*	*longissimum*		-	-
CGN16014	*Triticum*	*longissimum*		-	-
CGN13113	*Triticum*	*squarrosum*		-	-
CGN10734	*Triticum*	*squarrosum*		-	-
CGN16008	*Triticum*	*squarrosum*		-	-
CGN13119	*Triticum*	*squarrosum*		-	-
CGN13116	*Triticum*	*cylindricum*		-	-
CGN13117	*Triticum*	*cylindricum*		-	-
CGN10704	*Triticum*	*crassum*		+	-
CGN10706	*Triticum*	*crassum*		+	-
CGN10707	*Triticum*	*ventricosum*		-	-
CGN10712	*Triticum*	*ventricosum*		-	-
CGN6613	*Triticum*	*ovatum*		+	-
CGN10668	*Triticum*	*ovatum*		+	-
CGN10664	*Triticum*	*triaristatum*		+	-
CGN21070	*Triticum*	*triaristatum*		+	-
CGN10774	*Triticum*	*triunciale*		-	-
CGN6606	*Triticum*	*kotschyi*		+	-
CGN6607	*Triticum*	*columnare*		+	-
CGN6077	*Triticum*	*ventricosum*		-	-
CGN16015	*Triticum*	*lorentii*		+	-
CGN16016	*Triticum*	*caudatum*		-	-
CGN16017	*Triticum*	*peregrinum*		+	-
PI542175	*Aegilops*	*comosa*		-	-
PI551036	*Aegilops*	*comosa*		-	-
PI551038	*Aegilops*	*comosa*		-	-
PI551034	*Aegilops*	*comosa*		-	-
PI551032	*Aegilops*	*comosa*		-	-
PI551020	*Aegilops*	*comosa*		-	-
PI551080	*Aegilops*	*comosa*		-	-
PI551031	*Aegilops*	*comosa*		-	-
PI276970	*Aegilops*	*comosa*		-	-
PI551047	*Aegilops*	*comosa*		-	-

Supplementary Table 12. Durum and common wheat accessions evaluated for the presence of *Wtk1* and for resistance to *Pst*. Each accession was categorized with gene-specific diagnostic markers for the presence of the two conserved domains (Supplementary Table 9).

Germplasm number/variety number	genus	species	subspecies	KinI *Wtk1*	KinII *Wtk1*	Phenotype‡
280-1*	*Triticum*	*turgidum*	*durum*	+	+	R
280-2*	*Triticum*	*turgidum*	*durum*	+	+	R
Aristan†	*Triticum*	*turgidum*	*durum*	-	-	NT
Aziziah†	*Triticum*	*turgidum*	*durum*	-	-	NT
B10*	*Triticum*	*turgidum*	*durum*	+	+	R
B1*	*Triticum*	*turgidum*	*durum*	+	+	R
B2*	*Triticum*	*turgidum*	*durum*	+	+	R
B70*	*Triticum*	*turgidum*	*durum*	+	+	R
B9*	*Triticum*	*turgidum*	*durum*	+	+	R
Baio†	*Triticum*	*turgidum*	*durum*	-	-	NT
Bufala†	*Triticum*	*turgidum*	*durum*	-	-	NT
Capeiti8†	*Triticum*	*turgidum*	*durum*	-	-	NT
Cappelli†	*Triticum*	*turgidum*	*durum*	-	-	NT
D447	*Triticum*	*turgidum*	*durum*	-	-	S
Duilio†	*Triticum*	*turgidum*	*durum*	-	-	NT
Kronos	*Triticum*	*turgidum*	*durum*	-	-	S
Langdon	*Triticum*	*turgidum*	*durum*	-	-	S
Muri S 50 3†	*Triticum*	*turgidum*	*durum*	-	-	NT
Nursit 163	*Triticum*	*turgidum*	*durum*	-	-	S
Ofanto†	*Triticum*	*turgidum*	*durum*	-	-	NT
Pavone†	*Triticum*	*turgidum*	*durum*	-	-	NT
Razzak†	*Triticum*	*turgidum*	*durum*	-	-	NT
Roqueno†	*Triticum*	*turgidum*	*durum*	-	-	NT
Russello†	*Triticum*	*turgidum*	*durum*	-	-	NT
Sabil 1†	*Triticum*	*turgidum*	*durum*	-	-	NT
Santa†	*Triticum*	*turgidum*	*durum*	-	-	NT
Simeto†	*Triticum*	*turgidum*	*durum*	-	-	NT
Siyah Kilakli†	*Triticum*	*turgidum*	*durum*	-	-	NT
Svevo	*Triticum*	*turgidum*	*durum*	-	-	S
Taganrog†	*Triticum*	*turgidum*	*durum*	-	-	NT
Timilia†	*Triticum*	*turgidum*	*durum*	-	-	NT
Triminia†	*Triticum*	*turgidum*	*durum*	-	-	NT
Tripolino†	*Triticum*	*turgidum*	*durum*	-	-	NT
Vatan†	*Triticum*	*turgidum*	*durum*	-	-	NT
Villemur†	*Triticum*	*turgidum*	*durum*	-	-	NT
Waha†	*Triticum*	*turgidum*	*durum*	-	-	NT
Avocet S	*Triticum*	*aestivum*		-	-	S
Avocet+*Yr15**	*Triticum*	*aestivum*		+	+	R
BarNear	*Triticum*	*aestivum*		-	-	S
Baxter	*Triticum*	*aestivum*		-	-	S
Bobwhite	*Triticum*	*aestivum*		-	-	S
Bobwhite+*Yr15**	*Triticum*	*aestivum*		+	+	R
Chinese Spring	*Triticum*	*aestivum*		-	-	S
Combat	*Triticum*	*aestivum*		-	-	S
Corrigin	*Triticum*	*aestivum*		-	-	S
Corrigin+*Yr15**	*Triticum*	*aestivum*		+	+	R
Excalibur	*Triticum*	*aestivum*		-	-	S
Excalibur+*Yr15**	*Triticum*	*aestivum*		+	+	R
Fielder	*Triticum*	*aestivum*		-	-	S

Galil	*Triticum*	*aestivum*	-	-	S
Gedera	*Triticum*	*aestivum*	-	-	S
HSB 2408[*]	*Triticum*	*aestivum*	+	+	R
HSB 2527	*Triticum*	*aestivum*	-	-	S
HSB 2944[*]	*Triticum*	*aestivum*	+	+	R
HSB 2949[*]	*Triticum*	*aestivum*	+	+	R
HSB 3177[*]	*Triticum*	*aestivum*	+	+	R
Kulin	*Triticum*	*aestivum*	-	-	S
Kunlin+*Yr15*[*]	*Triticum*	*aestivum*	+	+	R
Merav	*Triticum*	*aestivum*	-	-	S
Mexico708	*Triticum*	*aestivum*	-	-	S
Ruby	*Triticum*	*aestivum*	-	-	S
Ruta	*Triticum*	*aestivum*	-	-	S
Sapphire	*Triticum*	*aestivum*	-	-	S
Sel07-97[*]	*Triticum*	*aestivum*	+	+	R
Sel20[*]	*Triticum*	*aestivum*	+	+	R
Sel32[*]	*Triticum*	*aestivum*	+	+	R
Sel4[*]	*Triticum*	*aestivum*	+	+	R
Sel46[*]	*Triticum*	*aestivum*	+	+	R
Sel7[*]	*Triticum*	*aestivum*	+	+	R
Shenton	*Triticum*	*aestivum*	-	-	S
Stilleto	*Triticum*	*aestivum*	-	-	S
Stilleto+*Yr15*[*]	*Triticum*	*aestivum*	+	+	R
Suncea	*Triticum*	*aestivum*	-	-	S
Suncea+*Yr15*[*]	*Triticum*	*aestivum*	+	+	R
UC1037	*Triticum*	*aestivum*	-	-	S
UC1037+*Yr15*[*]	*Triticum*	*aestivum*	+	+	R
UC1041	*Triticum*	*aestivum*	-	-	S
UC1041+*Yr15*[*]	*Triticum*	*aestivum*	+	+	R
UC1107	*Triticum*	*aestivum*	-	-	S
UC1107+*Yr15*[*]	*Triticum*	*aestivum*	+	+	R
UC1110	*Triticum*	*aestivum*	-	-	S
UC1110+*Yr15*[*]	*Triticum*	*aestivum*	+	+	R
UC1128	*Triticum*	*aestivum*	-	-	S
UC1128+*Yr15*[*]	*Triticum*	*aestivum*	+	+	R
UC1358	*Triticum*	*aestivum*	-	-	S
UC1358+*Yr15*[*]	*Triticum*	*aestivum*	+	+	R
UC1419	*Triticum*	*aestivum*	-	-	S
UC1419+*Yr15*[*]	*Triticum*	*aestivum*	+	+	R
UCKern	*Triticum*	*aestivum*	-	-	S
UCKern+*Yr15*[*]	*Triticum*	*aestivum*	+	+	R
V763[*]	*Triticum*	*aestivum*	+	+	R
Yoval	*Triticum*	*aestivum*	-	-	S
Zaher	*Triticum*	*aestivum*	-	-	S

[*]*Yr15* introgression lines from DIC G25[1].
[†]*T. durum* accessions from the Salamini collection[9].
[‡]Phenotypic response to inoculation with *Pst* isolate #5006 (R, resistant; S, susceptible; NT, not tested).

Supplementary Table 13. The presence of key conserved residues[4,10] in the putative kinase domains of the predicted proteins for 92 TKP sequences.

Name of the predicted protein*	Key conserved residues							
	G^{52}	K^{72}	E^{91}	H^{158}	H^{164}	D^{166}	N^{171}	D^{184}
TraesCS1A01G061500.1_K1	G	K	E	H	H	D	N	D
TraesCS1A01G197000.2_K1	G	K	E	H	H	D	N	D
TraesCS1A01G432400.1_K1	G	K	E	H	H	D	N	D
TraesCS1B01G079900.1_K1	G	K	E	H	H	D	N	D
TraesCS1D01G033500.1_K1	G	K	E	H	H	D	N	D
TraesCS2A01G510300.1_K1	G	K	E	H	H	D	N	D
TraesCS2B01G538000.1_K1	G	K	E	H	H	D	N	D
TraesCS2B01G538200.1_K1	G	K	E	H	H	D	N	D
TraesCS2D01G123700.1_K1	G	K	E	H	C	E	D	L
TraesCS2D01G124300.1_K1	G	K	E	H	C	E	D	L
TraesCS2D01G511500.1_K1	G	K	E	H	H	D	N	D
TraesCS2D01G579800.3_K1	G	K	E	H	H	D	N	D
TraesCS3B01G579200.1_K1	G	K	E	H	H	D	N	D
TraesCS4A01G334900.1_K1	G	K	E	H	H	D	N	D
TraesCS4A01G335000.1_K1	G	K	E	H	H	D	N	D
TraesCS5A01G161500.1_K1	G	K	E	H	H	D	N	D
TraesCS5A01G241300.2_K1	G	K	E	H	H	D	N	D
TraesCS5A01G449800.1_K1	G	K	E	H	H	D	N	D
TraesCS5B01G005400.3_K1	G	K	E	H	H	D	N	D
TraesCS5B01G159000.1_K1	G	K	E	H	H	D	N	D
TraesCS5B01G239600.1_K1	G	K	E	H	H	D	N	D
TraesCS5D01G166400.1_K1	G	K	E	H	H	D	N	D
TraesCS5D01G241800.1_K1	G	K	E	L	H	D	N	D
TraesCS5D01G247800.1_K1	G	K	E	H	H	D	N	D
TraesCS5D01G459500.1_K1	G	K	E	H	H	D	N	D
TraesCS5D01G459700.1_K1	G	K	E	H	H	D	N	N
TraesCS5D01G537200.1_K1	G	K	E	H	H	D	N	D
TraesCS6A01G020100.1_K1	G	K	E	H	H	D	N	D
TraesCS6A01G036400.1_K1	G	K	E	H	H	D	N	D
TraesCS6B01G029600.1_K1	G	K	E	H	H	D	N	D
TraesCS6B01G050800.1_K1	G	K	E	H	H	D	N	D
TraesCS6B01G050900.1_K1	G	K	E	H	H	D	N	D
TraesCS6B01G051000.1_K1	G	K	E	D	H	D	N	D
TraesCS6D01G025700.1_K1	G	K	E	H	H	D	N	D
TraesCS6D01G042200.1_K1	G	K	E	H	H	D	N	D
TraesCS6D01G042300.1_K1	G	K	E	H	H	D	N	D
TraesCS7B01G048900.1_K1	G	K	E	H	H	D	N	D
TraesCS7D01G147900.1_K1	G	K	E	H	H	D	N	D
WTK1_K1	G	K	E	H	H	D	N	D
MLOC_K1	G	K	E	H	H	D	N	D
RPG1_K2	G	K	E	H	H	D	N	D
HORVU1Hr1G011660.17_K1	G	K	E	H	H	D	N	D
HORVU1Hr1G051220.15_K1	G	K	E	H	H	D	N	D
HORVU5Hr1G050470.1_K1	G	K	E	H	H	D	N	D
HORVU5Hr1G107460.3_K1	G	K	E	H	H	D	N	D
HORVU6Hr1G003940.7_K1	G	K	E	H	H	D	N	D
HORVU6Hr1G025940.2_K1	G	K	E	H	H	D	N	D
HORVU7Hr1G001450.11_K2	G	K	E	H	H	D	N	D

HORVU7Hr1G001600.12_K2	G	K	E	H	H	D	N	D
Sc1Loc00250465.1_K1	G	K	E	H	H	D	N	D
Sc5Loc01920045.3_K1	G	K	E	Y	H	D	N	D
Sc2Loc00020948.6_K1	G	K	E	H	H	D	N	D
Os01t0310500-01_K1	G	K	E	H	H	D	N	D
Os07t0493200-01_K1	G	K	E	H	H	D	N	D
Os07t0493800-00_K1	G	K	E	H	H	D	N	D
Os07t0494300-00_K1	G	K	E	H	H	D	N	D
Os10t0141200-00_K1	G	K	E	H	H	D	N	D
Os10t0143866-00_K1	G	K	E	H	H	D	N	D
Os11t0173432-00_K1	G	K	E	H	H	D	N	D
Os11t0445300-01_K1	G	K	E	H	H	D	N	D
Os11t0553500-00_K1	G	K	E	H	H	D	N	D
Os11t0556400-00_K1	G	K	E	H	H	D	N	D
AQK57443.1_K2	G	K	E	H	H	D	N	D
AQK57450.1_K2	G	K	E	H	H	D	N	D
AQK57451.1_K1	G	K	E	H	H	D	N	D
AQK57454.1_K2	G	K	E	H	H	D	N	D
AQK58522.1_K1	G	K	E	H	H	N	N	D
AQK90211.1_K1	G	K	E	H	H	D	N	D
AQK92446.1_K1	G	K	E	H	H	D	N	D
ONM26931.1_K1	G	K	E	H	H	D	N	D
AT2G32800.1_K1	G	K	E	H	H	D	N	D
Pp1s31_26V6.1_K2	G	K	E	H	H	D	N	D
PGSC0003DMP400002294_K2	G	K	E	H	H	D	N	D
BnaA07g14690D_K1	G	K	E	H	H	D	N	D
BnaA09g41440D_K1	G	K	E	H	H	D	N	D
BnaC04g38500D_K1	G	Y	E	H	Y	D	N	D
BnaA03g15120D_K1	G	K	E	H	H	D	N	D
BnaA02g06510D_K1	G	K	E	H	H	D	N	D
Potri.017G055000_K1	G	K	E	H	H	D	N	D
Potri.001G315000_K1	G	K	E	H	H	D	N	D
SOBIC.010G171600.1.P_K1	G	K	E	H	H	D	N	D
SOBIC.005G096400.1.P_K1	G	K	E	H	H	N	N	D
SOBIC.008G022300.2.P_K1	G	K	E	H	H	D	N	D
SOBIC.001G353800.1.P_K1	G	K	E	H	H	D	N	D
SOBIC.008G148200.2.P_K1	G	K	E	H	H	D	N	D
SOBIC.010G028950.1.P_K1	G	K	E	H	H	D	N	D
SOBIC.005G154100.1.P_K1	G	K	E	H	H	D	N	D
SOBIC.009G246800.1.P_K1	G	K	E	H	H	D	N	D
SOBIC.005G155100.1.P_K1	G	K	E	H	H	D	N	D
SOBIC.005G154800.1.P_K1	G	K	E	H	H	D	N	D
SOBIC.005G060700.2.P_K1	G	K	E	H	H	D	N	D
SOBIC.001G354100.2.P_K1	G	K	E	H	H	D	N	G

*The predicted proteins are named according to the gene models in the respective genome assemblies. Suffix K1/2 marks the position of the putative kinase domain within the tandem kinase-pseudokinase structure relative to the 5'-end.

Supplementary Table 14. The presence of key conserved residues[4,10] in the putative pseudokinase domains of the predicted proteins for 92 TKP sequences.

Name of the predicted protein*	Key conserved residues							
	G^{52}	K^{72}	E^{91}	H^{158}	H^{164}	D^{166}	N^{171}	D^{184}
TraesCS1A01G061500.1_K2	S	N	K	H	H	N	K	D
TraesCS1A01G197000.2_K2	G	K	I	H	H	D	N	N
TraesCS1A01G432400.1_K2	G	R	E	N	C	N	N	D
TraesCS1B01G079900.1_K2	S	K	K	H	H	N	K	D
TraesCS1D01G033500.1_K2	G	S	S	H	H	N	T	D
TraesCS2A01G510300.1_K2	G	K	L	H	H	N	N	E
TraesCS2B01G538000.1_K2	G	K	H	H	H	N	N	E
TraesCS2B01G538200.1_K2	G	K	H	H	H	N	N	E
TraesCS2D01G123700.1_K2	G	Q	M	H	S	K	N	D
TraesCS2D01G124300.1_K2	G	K	D	H	S	K	N	D
TraesCS2D01G511500.1_K2	-	K	H	H	H	N	N	E
TraesCS2D01G579800.3_K2	G	K	E	H	H	D	N	G
TraesCS3B01G579200.1_K2	G	K	G	H	H	S	N	G
TraesCS4A01G334900.1_K2	S	K	E	H	H	D	S	G
TraesCS4A01G335000.1_K2	G	R	E	H	H	R	S	D
TraesCS5A01G161500.1_K2	L	K	E	H	H	N	A	S
TraesCS5A01G241300.2_K2	G	K	E	H	F	D	H	G
TraesCS5A01G449800.1_K2	G	K	E	H	P	D	N	E
TraesCS5B01G005400.3_K2	G	K	E	H	H	N	N	D
TraesCS5B01G159000.1_K2	L	K	E	H	H	N	A	S
TraesCS5B01G239600.1_K2	G	K	E	H	V	D	N	G
TraesCS5D01G166400.1_K2	L	K	E	H	H	N	A	S
TraesCS5D01G241800.1_K2	G	K	R	H	H	N	N	E
TraesCS5D01G247800.1_K2	G	K	E	H	V	D	N	D
TraesCS5D01G459500.1_K2	S	K	E	H	P	V	N	S
TraesCS5D01G459700.1_K2	A	K	K	H	P	V	N	A
TraesCS5D01G537200.1_K2	G	K	Q	H	H	D	N	D
TraesCS6A01G020100.1_K2	L	K	E	H	H	C	N	Y
TraesCS6A01G036400.1_K2	D	K	E	K	H	N	N	N
TraesCS6B01G029600.1_K2	G	K	E	H	H	G	N	W
TraesCS6B01G050800.1_K2	D	R	E	H	H	D	N	G
TraesCS6B01G050900.1_K2	D	R	E	H	H	N	N	D
TraesCS6B01G051000.1_K2	G	R	E	H	H	D	N	D
TraesCS6D01G025700.1_K2	G	K	E	H	H	G	N	W
TraesCS6D01G042200.1_K2	G	R	E	H	H	N	N	D
TraesCS6D01G042300.1_K2	D	R	E	H	H	D	N	D
TraesCS7B01G048900.1_K2	C	N	S	H	G	N	N	-
TraesCS7D01G147900.1_K2	C	N	S	H	G	N	N	G
WTK1_K2	S	K	K	H	H	N	K	G
MLOC_K2	G	K	E	H	H	D	N	D
RPG1_K1	G	K	E	H	H	E	K	G
HORVU1Hr1G011660.17_K2	D	K	E	H	H	N	N	G
HORVU1Hr1G051220.15_K2	G	K	I	H	H	D	N	N
HORVU5Hr1G050470.1_K2	L	K	E	H	H	N	A	S
HORVU5Hr1G107460.3_K2	G	K	E	H	P	D	N	K
HORVU6Hr1G003940.7_K2	D	K	E	H	H	G	S	Y
HORVU6Hr1G025940.2_K2	G	K	E	H	H	N	N	D
HORVU7Hr1G001450.11_K1	G	K	E	H	H	E	K	G

HORVU7Hr1G001600.12_K1	-	-	E	H	H	G	K	G
Sc1Loc00250465.1_K2	D	K	E	H	H	N	N	G
Sc5Loc01920045.3_K2	G	K	Q	H	H	G	N	-
Sc2Loc00020948.6_K2	G	K	H	H	H	N	N	E
Os01t0310500-01_K2	E	K	T	H	H	N	N	N
Os07t0493200-01_K2	C	K	E	H	H	N	N	G
Os07t0493800-00_K2	R	K	E	H	H	D	N	G
Os07t0494300-00_K2	C	K	E	H	H	D	D	G
Os10t0141200-00_K2	S	K	Q	H	-	S	D	K
Os10t0143866-00_K2	D	K	E	H	H	N	T	G
Os11t0173432-00_K2	G	K	E	H	H	D	N	D
Os11t0445300-01_K2	L	K	E	H	H	N	A	S
Os11t0553500-00_K2	D	K	E	H	H	P	D	C
Os11t0556400-00_K2	G	K	A	H	H	D	N	A
AQK57443.1_K1	-	-	-	H	H	N	N	D
AQK57450.1_K1	G	K	E	H	H	N	N	D
AQK57451.1_K2	G	R	E	H	-	M	-	-
AQK57454.1_K1	-	-	-	H	H	N	N	D
AQK58522.1_K2	G	K	E	-	-	-	-	-
AQK90211.1_K2	A	N	D	C	C	T	T	D
AQK92446.1_K2	L	K	E	H	H	S	A	S
ONM26931.1_K2	G	H	G	H	H	S	T	E
AT2G32800.1_K2	V	K	E	H	H	N	T	G
Pp1s31_26V6.1_K1	D	K	E	H	H	D	N	D
PGSC0003DMP400002294_K1	G	K	E	H	H	D	K	D
BnaA07g14690D_K2	G	K	E	H	H	D	N	D
BnaA09g41440D_K2	G	K	E	H	H	D	N	D
BnaC04g38500D_K2	G	K	K	H	Y	D	N	C
BnaA03g15120D_K2	V	K	E	H	H	N	T	G
BnaA02g06510D_K2	G	A	E	H	H	D	N	D
Potri.017G055000_K2	V	K	E	H	H	N	S	N
Potri.001G315000_K2	V	K	E	H	H	N	S	N
SOBIC.010G171600.1.P_K2	G	K	E	H	H	D	N	D
SOBIC.005G096400.1.P_K2	G	K	E	H	H	D	N	D
SOBIC.008G022300.2.P_K2	D	R	E	C	H	N	C	I
SOBIC.001G353800.1.P_K2	G	I	E	H	Y	N	D	D
SOBIC.008G148200.2.P_K2	D	K	D	H	H	N	N	E
SOBIC.010G028950.1.P_K2	G	V	T	Q	H	N	-	A
SOBIC.005G154100.1.P_K2	G	H	E	H	H	S	T	E
SOBIC.009G246800.1.P_K2	L	K	D	H	H	N	A	S
SOBIC.005G155100.1.P_K2	G	R	E	H	H	Y	K	G
SOBIC.005G154800.1.P_K2	G	R	E	H	H	S	T	E
SOBIC.005G060700.2.P_K2	G	K	E	H	H	N	N	D
SOBIC.001G354100.2.P_K2	G	A	E	H	H	N	N	D

*The predicted proteins are named according to the gene models in the respective genome assemblies. Suffix K1/2 marks the position of the putative pseudokinase domain within the tandem kinase-pseudokinase structure relative to the 5'-end.

Supplementary References:

1. Yaniv, E. *et al*. Evaluation of marker-assisted selection for the stripe rust resistance gene *Yr15*, introgressed from wild emmer wheat. *Mol. Breeding* **35**, 43 (2015).
2. Raats, D. *et al*. The physical map of wheat chromosome 1BS provides insights into its gene space organization and evolution. *Genome Biol.* **14**, R138 (2013).
3. Zuckerkandl, E. & Pauling, L. "Evolutionary divergence and convergence in proteins" in *Evolving Genes and Proteins*, V. Bryson, H. J. Vogel, Eds. (Academic Press, New York. 1965), pp. 97–166.
4. Hanks, S. K., Quinn, A. M. & Hunter, T. The protein kinase family: conserved features and deduced phylogeny of the catalytic domains. *Science* **241**, 42–52 (1988).
5. Zulawski, M. *et al.* The *Arabidopsis* Kinome: phylogeny and evolutionary insights into functional diversification. *BMC Genomics* **15**, 548 (2014).
6. Raats, D. *et al*. "Application of CAPS markers for genomic studies in wild emmer wheat" in *Cleaved Amplified Polymorphic Sequences (CAPS) Markers in Plant Biology,* Y. Savrukov, Ed. (Nova Science Publishers, New York. 2014), pp. 31–61.
7. Paux, E. *et al*. *Plant biotechnology journal* **8**,196–210 (2010).
8. Nevo, E., Korol, A.B., Beiles, A. & Fahima, T. *Evolution of Wild Emmer and Wheat Improvement: Population Genetics, Genetic Resources, and Genome Organization of Wheats Progenitor, Triticum dicoccoides*. (Springer, Berlin, 2002).
9. Özkan, H. *et al*. A reconsideration of the domestication geography of tetraploid wheats. *Theor. Appl. Genet*. **110**, 1052–1060 (2005).
10. Kannan, N., Taylor, S. S., Zhai, Y., Venter, J. C. & Manning, G. Structural and functional diversity of the microbial kinome. *PLoS Biology* **5**, 467–478 (2007).
